# Subnational estimation of modern contraceptive prevalence in five sub-Saharan African countries: a Bayesian hierarchical approach

**DOI:** 10.1186/s12889-019-6545-3

**Published:** 2019-02-20

**Authors:** Qingfeng Li, Thomas A. Louis, Li Liu, Chenguang Wang, Amy O. Tsui

**Affiliations:** 1 Department of International Health, Johns Hopkins Bloomberg School of Public Health, Baltimore, MD USA; 2 Department of Population, Family and Reproductive Health, Johns Hopkins Bloomberg School of Public Health, Baltimore, MD USA; 3 Department of Biostatistics, Johns Hopkins Bloomberg School of Public Health, Baltimore, MD USA; 4 Sidney Kimmel Comprehensive Cancer Center, Johns Hopkins University, Baltimore, MD USA

**Keywords:** Family planning, Bayesian hierarchical model, Subnational estimates, Sub-Saharan Africa

## Abstract

**Background:**

Global monitoring efforts have relied on national estimates of modern contraceptive prevalence rate (mCPR) for many low-income countries. However, most contraceptive delivery programs are implemented by health departments at lower administrative levels, reflecting a persisting gap between the availability of and need for subnational mCPR estimates.

**Methods:**

Using woman-level data from multiple semi-annual national survey rounds conducted between 2013 and 2016 in five sub-Saharan African countries (Burkina Faso, Ethiopia, Ghana, Kenya, and Uganda) by the Performance, Monitoring and Accountability 2020 project, we propose a Bayesian Hierarchical Model with a standard set of covariates and temporally correlated random effects to estimate the level and trend of mCPR for first level administrative divisions in each country.

**Results:**

There is considerable narrowing of the uncertainty interval (UI) around the model-based estimates, compared to the estimates directly based on the survey data. We find substantial variations in the estimated subnational mCPRs. Uganda, for example, shows a gain in mCPR of 6.4% (95% UI: 4.5–8.3) based on model estimates of 20.9% (19.6–22.2) in mid-2014 and 27.3% (26.0–28.8) in mid-2016, with change across 10 regions ranging from − 0.6 points in Karamoja to 9.4 points in Central 2 region. The lower bound of the UIs of the change over four rounds was above 0 in 6 regions. Similar upward trends are observed for most regions in the other four countries, and there is noticeable within-country geographic variation.

**Conclusions:**

Reliable subnational estimates of mCPR empower health departments in evidence-based policy making. Despite nationally increasing mCPRs, regional disparities exist within countries suggesting uneven contraceptive access. Raising investments in disadvantaged areas may be warranted to increase equity in access to modern contraceptive methods.

**Electronic supplementary material:**

The online version of this article (10.1186/s12889-019-6545-3) contains supplementary material, which is available to authorized users.

## Background

Contraceptive-assisted birth spacing and limiting, also known as family planning, is a key component of primary and reproductive health care, with multiple health and development benefits to women, children, and society [1]. Contraceptive practice is associated with lower maternal mortality [2], greater women’s empowerment [3, 4], better female schooling [5], improved child health [6] and increased household income [7]. Universal access to sexual and reproductive health-care services, including family planning, information and education, and the integration of reproductive health into national strategies and programs by 2030 is a defined target (Target 3.7) in the Sustainable Development Goals (SDGs) [8]. Through the Family Planning 2020 (FP2020) initiative more than 30 governments from low-income countries have pledged to expand contraceptive service access and help satisfy women’s needs for modern contraception (see familyplanning2020.org). To track the progress toward the 2030 SDG target and FP2020 goal of adding 120 million modern contraceptive users in the world’s 69 poorest countries by 2020, quality data on key family planning indicators are needed at both national and subnational levels.

There have been major gains over the past five decades to measure demographic and health indicators in low-income countries with standardized data collection procedures, relying heavily on population-level surveys and enabling public data access. Recent national estimates of modern contraceptive prevalence rates (mCPR) among all or married women of childbearing age are available for many low-income countries with a high level of precision (e.g. a margin of error of 2 percentage points) [9, 10]. However, these national survey-based estimates are not available at frequent intervals and largely depend on the availability of international funding. For local monitoring and evaluation, this periodicity and national focus is impractical as most large-scale interventions are planned and implemented over short program cycles by health departments in administrative units below the national level. Cross-national estimates of mCPR serve global interests but not those of local policy makers. Regional, county and district officials require estimates of indicators to monitor and evaluate progress at their levels of operations and responsibilities. Health management information systems are gradually being strengthened but their coverage and accuracy suffer from incomplete reporting, especially of private sector contributions, weak data integration and insufficient resources for optimal functioning. The lack of quality data and regular and frequent estimates of key indicators at the subnational level have hindered health and development authorities’ efforts to strengthen local systems’ delivery of contraception and other reproductive health care services. Contraceptives and other procured health commodities experience stock outs which are important to monitor and meet client demand for health care in a timely manner. Ensuring that data and indicator estimates are available to subnational government units to inform their implementation is necessary for effective delivery of family planning to local communities.

One primary reason for the lack of subnational indicators is the high cost of increasing the household sample sizes of national surveys to generate estimates at lower levels with acceptable precision. Resource constraints in low-income countries are unlikely to be resolved in the near future, while the information requirements in these places will grow in scale and urgency. There are alternative approaches to filling the information gaps in the short term, one of which is to generate subnational estimates using appropriate statistical models.

Small area estimation techniques have been widely used to improve estimates for sub-areal domains, such as states, provinces or other local communities [11–13]. A sample area or domain is considered to be small if the area-specific sample is not large enough to support direct estimates (usually based on a maximum likelihood estimator) with adequate precision [14]. Of special note is that small refers to the size of the sample, not of the population. Whether a sample is considered to be large or small is associated with the uncertainty of the estimate and related to sample size but not identical to it. For a survey designed to generate national estimates, a region or province can be a small area if the underlying sample estimate is uncertain, even though the area’s geography or population size may be large.

Small area estimates can be useful for monitoring the progress of multiple SDG goals at the subnational level. Equity in SDG achievement and inclusion of all persons are reflected in the definitions of many targets, such as target 3.7 mentioned above. However, even while tracking national-level progress, individuals belonging to certain subgroups, defined by socioeconomic status, religion, culture or geographic location, may be inadequately represented in standard measurements. Small area estimation can generate reliable estimates for these subgroups to allow improved monitoring of trends and evaluation and accountability of support programs.

Multiple types of statistical models have been employed to generate small area estimates, such as linear mixed models [15] and multi-level random-intercept models [16]. Other studies have shown that using the Bayesian approach to account for variation and association within- and across-areas has comparative advantages in stabilizing small area estimates [17, 18]. The Bayesian approach has been applied to estimate family planning indicators. For example, a recent article adapted a Bayesian Hierarchical model originally developed for national and global estimates using a series of household surveys to generate subnational estimates of several key family planning indicators for 29 states and union territories in India [19].

The present study makes two contributions to the field. First, our model includes a comprehensive set of explanatory covariates that have been shown in the literature to be associated with modern contraceptive use. We consider failure to leverage such covariates a limitation of the previous Bayesian models for family planning outcomes [20, 21]. Second, we model contraception using woman-level outcome and explanatory indicators while previous work relied on Bayesian modelling at the aggregate level (state, national, regional, and global). By utilizing rich information available for individual women and by avoiding the loss of information due to aggregation, our model is expected to achieve better predictive performance and consequently generate more stable and accurate estimates for small areas. Studies using both real data and simulated data have found that woman-level models were consistently more accurate than aggregate-level models [22, 23].

The objective of the present study is to build a Bayesian hierarchical model to estimate the levels and trends of mCPR for first-level administrative divisions in five sub-Saharan African countries with national survey data collected by the Performance Monitoring and Accountability 2020 (PMA2020) project [17]. In the following sections, we first introduce the data source and methods, and then present the main results. In the discussion section we comment on the intrinsic benefits of statistical modeling for small area estimation, particularly using PMA2020 data.

## Methods

### Data

The data analyzed in this study are drawn from national PMA2020 surveys with multiple rounds [17, 24]. Funded by the Bill and Melinda Gates Foundation, PMA2020 was originally designed to facilitate annual progress reporting in support of the goals and principles of FP2020 across priority countries in Africa and Asia. It uses mobile devices (smartphones) to routinely gather nationally representative data on key family planning indicators. Data are collected at the woman, household, and facility levels by a network of resident enumerators stationed throughout the country. By using GPS-enabled mobile devices, the enumerators are able to collect accurate geographic information for each household. The survey platform can be integrated into national monitoring and evaluation systems at low cost with rapid turnaround and can be utilized for other areas of data collection and measurement, as well as linked to administrative records.

PMA2020 surveys are nationally representative in all five countries. This study capitalizes on the four survey rounds (R1 to R4) of PMA2020 conducted in Ethiopia, Ghana, Kenya, and Uganda between September 2013 and May 2016 at semi-annual intervals. Burkina Faso increased its sample sizes for Rounds 3 and 4, which are those used in this analysis. Additional details on the PMA2020 surveys, including participation rates, are reported in Zimmerman et al. [25] See Additional file 1 (p 15) for additional details about PMA2020 survey design.

### Statistical analysis

**Fig. 1:**
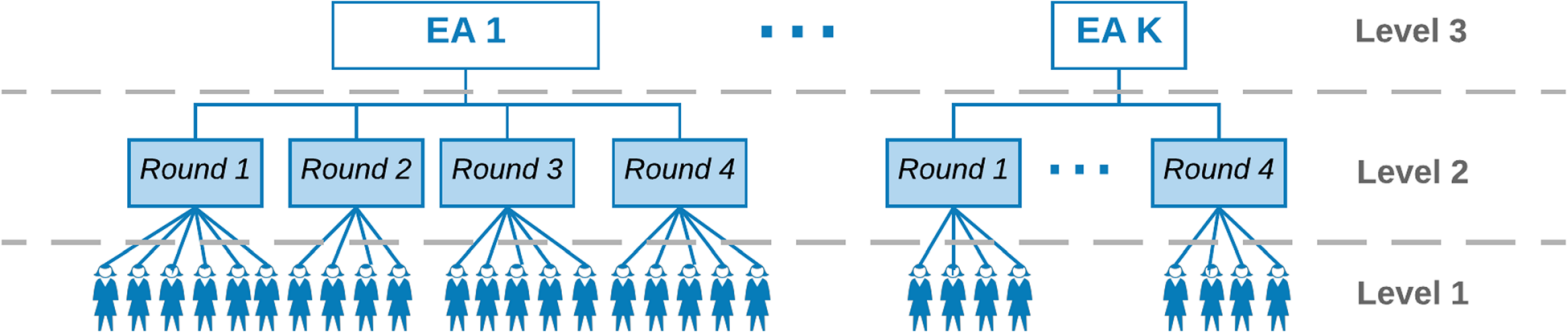
Statistical structure of the Bayesian hierarchical model

Our analytical data have a hierarchical structure with individual women as level 1, round as level 2, and EA as level 3. Accordingly, we proposed a hierarchical model to reflect the structure of the data (Fig. 1).

Bayesian computations are more complicated than standard logistic regression in the frequentist approach, but the corresponding degree of complexity is necessary for this study to respect the hierarchical structure of the data and stabilize the estimates, thereby reducing regression-to-the-mean effects and accounting for temporal variation in area-specific estimates. Bayesian methods support stabilization of small area estimates by shrinkage toward a regression surface, with shrinkage ranging from minimal for areas with many cases to substantial for areas with very few cases. Therefore, it accommodates a range of plausible alternatives between the extremes of using a regression equation for estimation versus using direct estimates. Moreover, the approach carries forward all uncertainties, those in estimating the regression surface, the between-area variance, and the sampling variance of the direct estimates. The full posterior distribution that reflects all uncertainty is particularly powerful in addressing questions that are hard in the traditional logistic regression. For example, if there are targets for the indicator of interest, the Bayesian model can be used to predict the probability of achieving that target, given the observed data and their associated uncertainty. This is usually less straightforward in the frequentist approach.

Our model improves upon a published Bayesian model [19] by including a series of proximate and distal determinants of family planning. The information collected in a standard fashion through the PMA2020 platform makes this possible. Since most covariates in the model vary over women within an area, woman-level data are used in this study. After the model estimation, we aggregate the woman-level estimates to the desired higher level, such as region. The model is specified below:

1 $$ logit\left({P}_{ikt}\right)={X}_{ikt}\beta +{u}_{kt} $$

Where *k* = 1, … , *K* indexes areas; *i* = 1, … , *n*_*kt*_ indexes women in area *k*; *n*_*kt*_ is the number of women in area *k* in round *t*; *t* = 1, . . , *T* indexes rounds; *Y*_*ikt*_ = 0/1 is an indicator of woman *i* in area *k* at round *t* not using (0) or using [1] a modern contraceptive method; [*Y*_*ikt*_| *P*_*ikt*_]~*Bernoulli*(*P*_*ikt*_); *P*_*ikt*_ is the true underlying probability of using modern contraceptive method for woman *i* in area *k* in round *t* conditional on [*X*_*ikt*_, *β*, *u*_*kt*_]; *X*_*ikt*_ is the vector of covariates for woman *i* in area *k* in round *t* including round-specific intercepts; *β* is the vector of regression coefficients; *U*_*kt*_ is the random effect for area *k* in round *t*. Figure 1 illustrates the statistical structure of the BHM. The direct (maximum likelihood estimate, MLE) estimate is the number of users divided by EA sample size of women of reproductive age.

We consider the autoregressive process of order one (AR1) structure for the random effects *u*_*kt*_ to capture the temporal correlation across rounds [26]. This characterization captures the serial correlation in the influences of the area-level unobserved factors.

Based on theory, a review of previous empirical studies, and model assessment, we arrived at a list of 12 covariates: residence, schooling, wealth quintile, child survival, age, cohabitation, recent sex, health worker visit, family planning message, fertility intention, parity, and distance to the nearest facility. See the Additional file 1 for their definitions (p 16). The Additional file 1 (pp 1–14) includes details of the model specification, covariate selection, diagnosis, and post-estimation prediction.

## Results

Table 1 profiles the PMA2020 surveys in the five countries. Ghana’s four survey rounds covered all 10 regions in the country, interviewing a total of 16,157 women of reproductive ages (WRAs) from 94 EAs. Ethiopia’s survey areas covered 11 regions in the country with a total of 24,237 WRAs interviewed across a subsample of 183 EAs consistently present in the four rounds. The PMA2020 sample in Kenya covered 17,242 WRAs across 120 EAs in the four rounds. Uganda’s four survey rounds interviewed 14,567 WRAs from 109 EAs. Uganda’s subnational governance structure is at the district level but this study has adopted the classification of ten regions used by the Uganda Demographic and Health Survey for level-1 estimation [27]. The PMA2020 sample in Burkina Faso was substantially expanded in round 3, and therefore this study only uses data from round 3 and 4. Those two rounds covered all of the country’s 13 regions with a total of 6392 WRAs in 83 EAs. The compositional characteristics of the study sample are provided in Additional file 1 (pp 4–13).

**Table 1:** Description of PMA2020 surveys in Ghana, Ethiopia, Kenya, Uganda and Burkina Faso

Survey	Month/Year of fieldwork	# of regions	# of enumeration areas	# of women
Ghana				
Round 1	Sep-Nov 2013	10	94	3460
Round 2	Mar-May 2014	10	94	3645
Round 3	Sep-Nov 2014	10	94	4251
Round 4	May-Jul 2015	10	94	4801
Ethiopia				
Round 1	Jan-Mar 2014	11	183	5849
Round 2	Oct-Dec 2014	11	183	6013
Round 3	Apr-May 2015	11	183	6210
Round 4	Mar-Apr 2016	11	183	6165
Kenya				
Round 1	May-Jul 2014	9	120	3729
Round 2	Nov-Dec 2014	9	120	4304
Round 3	Jun-Jul 2015	9	120	4364
Round 4	Nov-Dec 2015	9	120	4845
Uganda				
Round 1	May-Jun 2014	10	109	3672
Round 2	Jan-Feb 2015	10	109	3562
Round 3	Aug-Sep 2015	10	109	3628
Round 4	Mar-Apr 2016	10	109	3705
Burkina Faso				
Round 3	Mar-May 2016	13	83	3225
Round 4	Nov 2016-Jan2017	13	83	3167

Notes:

(1) Only enumeration areas covered in all four rounds (or two for Burkina Faso) are included in the analytical sample

(2) In this study region denotes the first-level administrative division under the national one. The exact name/definition varies across countries. It is called region in Ethiopia, Ghana, Uganda, and Burkina Faso and county in Kenya

(2) Ethiopia, Ghana, Uganda and Burkina Faso PMA2020 surveys covered all regions in the country; Kenya PMA2020 surveys covered 9 counties, selected from 47 semi-autonomous counties using probability proportional to size (PPS) approach

The PMA2020 sample size is by design sufficient to produce accurate and reliable estimates of mCPR at the national level and separately for rural/urban areas, but not for level-1 units, except Kenya and large regions in Ethiopia. We built a Bayesian hierarchical model (BHM) to generate level-1 estimates whose reliability and accuracy can be evaluated with standardized residuals and shrinkage plots.

Table 2 presents the round-specific Bayesian estimates of mCPR by region for each of the five countries, along with the uncertainty intervals (UI, i.e. 95% credible interval from Bayesian posterior distribution). The direct estimates are available in the Additional file 1 (pp 14–15). There is a clear pattern of narrowing UIs for the regional estimates. For example, in Ashanti region in Ghana, the Round 1 direct estimate of mCPR is 16.1% and the BHM estimate is 15.9%. The length of the UI is 5.0 percentage points (13.7–18.7) for the former and 4.1 percentage points (14.0–18.1) for the latter. In Ethiopia, the region of Benishangul Gumuz has a UI of 4.3–21.7 around a Round 1 direct estimate of 10.9% compared to 12.2–19.3 around the BHM estimate of 15.4%. By Round 4, the BHM estimate is 16.3% and the width of the UI narrows to 6.4 percentage points (13.2–19.6), as compared to 7.1 percentage points for Round 1. For the same period, the width of the UI for direct estimate only slightly decreased from 17.4 in round 1 to 15.5 percentage points in round 4. The notable difference in the width of UI indicates improved accuracy through accounting for those 12 covariates and temporally correlated random effects in the BHM.

**Table 2:** Bayesian estimates of the modern contraceptive prevalence rate and 95% uncertainty intervals in Ghana, Ethiopia, Kenya, Uganda and Burkina Faso by round

Country	Region	Round 1			Round 2			Round 3			Round 4		
		mCPR	Lower	Upper	mCPR	Lower	Upper	mCPR	Lower	Upper	mCPR	Lower	Upper
Ghana	Ashanti	15.9	14.0	18.1	16.5	14.6	18.8	18.1	15.8	20.6	23.0	20.5	25.9
	Brong Ahafo	16.1	13.2	19.1	15.2	12.3	18.1	22.6	19.0	26.5	24.0	20.4	27.8
	Central	16.5	13.6	19.9	19.7	16.4	23.2	21.7	18.2	25.6	24.8	21.2	28.9
	Eastern	13.5	10.9	16.7	13.6	10.8	16.6	18.0	15.3	21.2	23.6	19.9	27.7
	Greater Accra	15.2	12.7	17.5	15.8	13.6	18.1	19.3	17.0	21.7	23.2	20.7	25.9
	Northern	8.0	6.4	9.8	6.3	5.0	7.8	10.8	8.4	13.9	13.6	11.2	16.7
	Upper East	17.4	13.8	21.7	16.2	12.8	19.9	19.3	14.5	25.4	30.6	24.2	36.9
	Upper West	24.6	19.5	30.7	20.1	16.0	24.6	25.5	20.9	31.0	32.6	26.7	39.9
	Volta	9.4	7.2	12.2	8.2	6.4	10.4	14.5	11.8	17.2	16.7	14.3	19.7
	Western	7.5	5.4	9.8	8.7	6.9	10.8	13.0	10.5	16.0	13.9	11.2	16.9
	ALL	14.0	12.8	15.1	14.0	12.9	15.0	18.3	17.1	19.5	22.6	21.3	23.9
Ethiopia	Addis Ababa	21.8	19.9	23.8	22.9	21.1	24.9	28.3	26.4	30.3	26.8	24.8	29.0
	Afar	5.4	3.5	7.7	11.6	7.9	16.1	18.2	12.8	23.8	12.9	8.9	17.7
	Amhara	33.8	31.7	35.7	34.7	32.9	36.7	33.5	31.6	35.2	32.9	31.1	34.6
	Benishangul Gumuz	15.4	12.2	19.3	11.3	9.1	14.2	15.5	12.7	18.5	16.3	13.2	19.6
	Dire Dawa	16.3	11.2	22.0	22.0	16.4	28.6	29.6	24.4	35.0	37.4	29.8	44.8
	Ethiopia Somali	9.2	6.3	12.5	7.6	5.3	10.2	8.1	5.8	10.7	8.3	5.7	11.3
	Gambella	26.2	21.1	31.1	21.8	17.5	27.4	21.8	17.4	26.4	26.4	20.8	33.6
	Harari	26.8	20.3	33.9	18.0	13.5	22.8	23.4	18.1	29.8	24.6	18.8	31.4
	Oromiya	18.9	16.9	20.9	20.0	18.3	21.7	23.3	21.7	24.9	22.4	20.7	24.2
	SNNPR	22.3	20.6	24.0	24.2	22.4	26.1	27.4	25.8	29.2	27.7	25.8	29.8
	Tigray	20.2	18.3	22.1	22.4	20.6	24.1	23.5	21.7	25.3	22.4	20.6	24.4
	ALL	23.0	21.9	24.1	24.2	23.1	25.3	27.2	26.2	28.2	26.6	25.5	27.6
Kenya	Bungoma	42.3	39.4	45.2	39.2	36.7	41.5	44.1	41.6	46.7	44.4	41.6	46.9
	Kericho	40.1	36.4	43.8	37.9	34.8	40.9	43.1	40.0	46.6	40.5	37.4	43.7
	Kiambu	41.2	37.1	45.3	44.4	40.9	48.0	47.9	44.8	51.2	50.0	46.0	53.7
	Kilifi	29.9	26.9	33.2	26.4	23.7	29.3	32.9	29.8	35.9	32.8	30.0	36.0
	Kitui	41.0	37.5	44.1	39.7	37.1	42.4	50.2	47.3	52.8	50.7	47.8	53.8
	Nairobi	43.8	39.6	47.9	42.1	38.7	45.2	51.4	47.5	55.0	49.2	45.4	53.0
	Nandi	44.2	41.1	47.3	45.0	42.4	47.8	47.9	45.5	50.4	47.2	44.4	50.0
	Nyamira	50.5	47.5	53.7	50.6	47.9	53.3	57.0	54.1	60.0	55.6	52.8	58.3
	Siaya	41.7	38.1	45.2	40.1	37.1	43.2	46.9	43.8	49.6	49.3	46.1	52.4
	ALL	41.4	39.8	42.9	40.2	38.9	41.5	46.7	45.3	48.1	46.1	44.7	47.4
Uganda	Central1	26.7	22.8	30.9	31.6	27.7	36.0	32.0	28.1	36.3	30.7	26.7	35.0
	Central2	20.2	17.3	23.3	27.6	24.0	31.0	29.6	26.2	32.9	29.6	26.3	33.2
	East Central	18.0	15.7	20.5	24.2	21.5	27.1	25.2	22.2	28.2	26.4	23.7	29.5
	Eastern	19.3	16.4	22.1	24.4	21.4	27.3	23.8	20.9	26.7	26.6	23.4	29.8
	Kampala	30.0	26.7	33.1	37.5	34.5	40.9	37.5	34.5	40.7	35.8	32.6	38.8
	Karamoja	9.0	6.1	13.1	7.8	5.5	11.0	6.7	4.4	10.2	8.4	5.7	12.2
	North	22.0	19.2	25.1	25.6	23.0	28.4	23.3	20.7	26.1	26.2	23.1	29.3
	South West	22.6	20.2	25.2	26.8	24.2	29.5	25.5	23.0	28.1	30.2	27.0	33.0
	West Nile	9.7	7.5	12.1	14.9	12.1	17.9	14.5	11.8	17.4	16.3	13.6	19.3
	Western	25.1	21.7	28.2	30.3	26.6	33.9	28.0	24.5	31.6	29.4	25.9	33.3
	ALL	20.9	19.6	22.2	26.1	24.8	27.5	25.8	24.5	27.2	27.3	26.0	28.8
Burkina Faso	Boucle du Mouhoun							19.7	15.9	24.1	19.8	16.1	24.1
	Cascades							24.9	20.5	29.3	20.5	16.2	25.2
	Centre							31.8	28.8	35.1	34.4	31.1	37.8
	Centre Est							14.5	10.9	18.4	18.2	14.4	22.6
	Centre Nord							18.3	14.5	22.1	16.2	12.5	20.1
	Centre Ouest							20.9	16.7	26.2	19.8	15.5	24.6
	Centre Sud							24.5	17.6	32.1	24.3	16.7	32.8
	Est							18.7	15.5	22.3	23.2	19.3	27.6
	Haut Bassins							27.8	23.8	31.7	29.2	25.5	33.1
	Nord							20.2	16.5	24.9	19.5	15.3	23.5
	Plateau Central							23.4	17.2	30.2	16.0	11.4	21.3
	Sahel							12.7	8.7	17.4	13.6	10.1	17.8
	Sud Ouest							16.0	11.7	21.5	15.5	10.9	21.7
	ALL							21.5	20.2	22.9	21.8	20.5	23.4

Note: Lower and upper denote the boundaries of the 95% uncertainty interval from Bayesian posterior distribution

The wide uncertainty intervals of the direct estimates for lower-level regions with PMA2020 data make it difficult to detect statistically significant change in indicator trends. Traditional tests (e.g. t-test, chi-squared test) are unable to incorporate the serial correlation of the estimates from different rounds. As discussed above, the full posterior distribution captures all the information including uncertainties in the estimates and enables us to rigorously examine temporal change. We can obtain the full distribution of the difference in mCPR between two rounds. In this study, we are particularly interested in how the subnational mCPR has changed from round 1 to round 4 (in approximately two years).

**Fig. 2:**
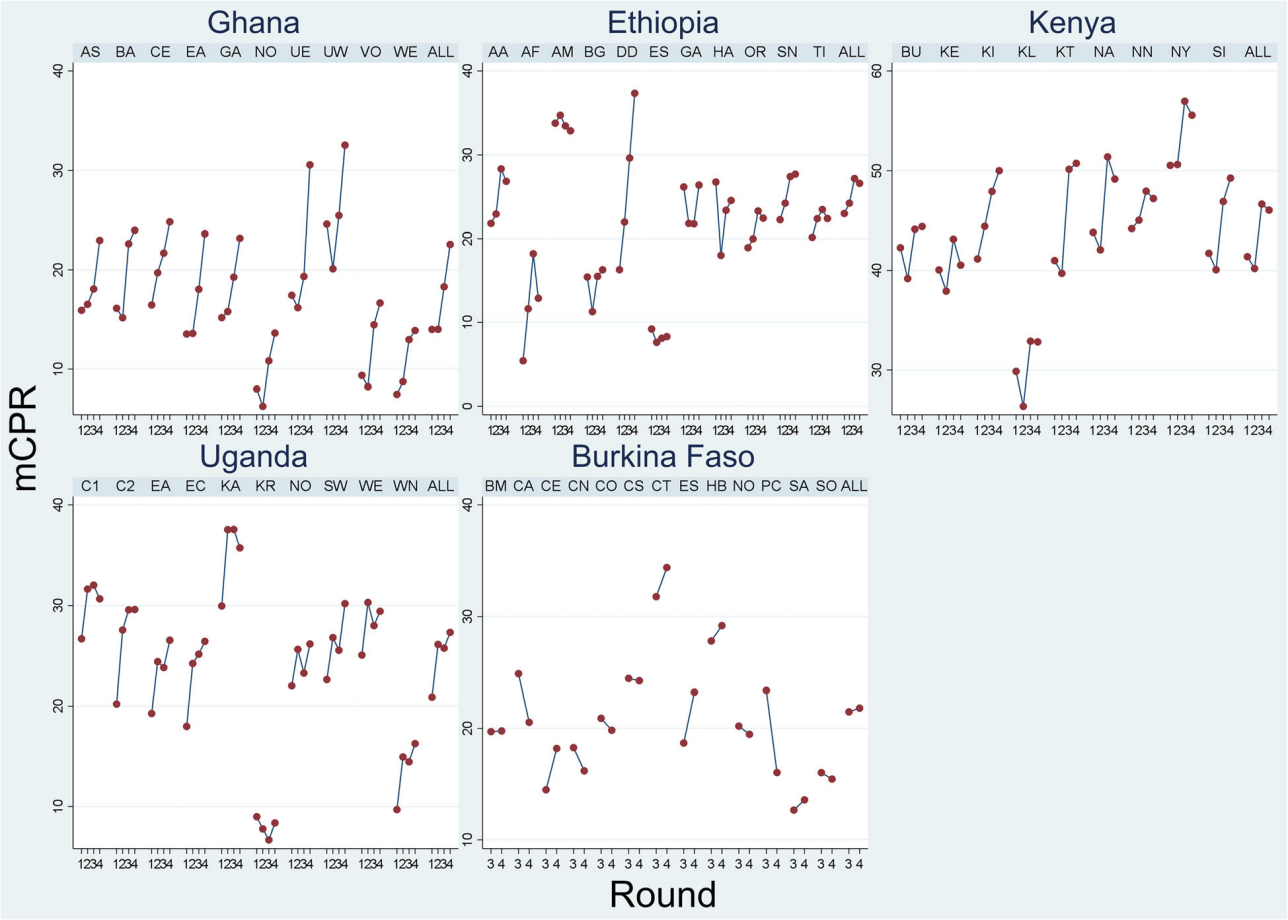
Bayesian estimates of trends in modern contraceptive prevalence rate (mCPR) by region and PMA2020 round. Note: See Table 3 for the full name of regions; ALL denotes the national estimate

Table 3 shows the subnational distributions of temporal change for in the five countries, while Fig. 2 graphs the region- and round-specific trends in Bayesian mCPR estimates. Overall, similar to the substantial variation in mCPR observed at the regional level, subnational progress also varies visibly across the five countries.

**Table 3:** Bayesian estimates of temporal change in modern contraceptive prevalence: Rounds 1 to 4

Country	Region	Change in mCPR	Uncertainty Interval
Ghana	Ashanti (AS)	7.0	3.7–10.4
	Brong Ahafo (BA)	7.9	3.0–13.0
	Central (CE)	8.3	3.2–13.4
	Eastern (EA)	9.9	5.8–15.0
	Greater Accra (GA)	8.0	4.7–11.6
	Northern (NO)	5.6	2.6–9.1
	Upper East (UE)	12.8	5.9–20.7
	Upper West (UW)	7.8	−0.3 - 16.5
	Volta (VO)	7.4	3.7–10.9
	Western (WE)	6.4	3.1–9.8
	ALL	8.6	6.8–10.4
Ethiopia	Addis Ababa (AA)	5.0	2.2–7.8
	Afar (AF)	7.4	3.6–12.2
	Amhara (AM)	−0.9	−3.6 - 1.8
	Benishangul Gumuz (BG)	0.8	−3.8 - 5.0
	Dire Dawa (DD)	21.1	12.8–29.7
	Ethiopia Somali (ES)	−0.9	−4.7 - 2.9
	Gambella (GA)	0.4	−6.9 - 8.1
	Harari (HA)	−2.3	−10.4 - 6.3
	Oromiya (OR)	3.6	1.0–6.1
	SNNPR (SN)	5.4	3.0–8.0
	Tigray (TI)	2.3	−0.4 - 4.8
	ALL	3.6	2.0–5.2
Kenya	Bungoma (BU)	2.2	−2.0 - 6.0
	Kericho (KE)	0.6	−4.5 - 4.8
	Kiambu (KI)	8.8	3.7–14.1
	Kilifi (KL)	3.0	−1.5 - 7.1
	Kitui (KT)	9.8	5.7–14.4
	Nairobi (NA)	5.3	0.2–10.5
	Nandi (NN)	3.0	−1.0 - 7.0
	Nyamira (NY)	5.0	0.9–9.3
	Siaya (SI)	7.5	3.1–12.2
	ALL	4.7	2.7–6.8
Uganda	Central1 (C1)	4.0	−1.7 - 9.6
	Central2 (C2)	9.4	5.1–14.0
	East Central (EC)	8.6	4.8–12.3
	Eastern (EA)	7.3	3.1–11.5
	Kampala (KA)	5.8	1.3–10.2
	Karamoja (KR)	−0.6	−5.2 - 3.9
	North (NO)	4.2	− 0.1 - 8.1
	South West (SW)	7.5	3.8–11.7
	West Nile (WN)	6.5	3.0–10.1
	Western (WE)	4.5	−0.2 - 9.2
	ALL	6.4	4.5–8.3
Burkina Faso	Boucle du Mouhoun (BM)	0.1	−5.3 - 5.7
	Cascades (CA)	−4.4	−10.0 - 1.6
	Centre (CT)	2.7	−1.6 - 6.9
	Centre Est (CE)	3.7	−1.1 - 8.8
	Centre Nord (CN)	−2.1	−6.9 - 2.8
	Centre Ouest (CO)	−1.1	−7.4 - 4.9
	Centre Sud (CS)	−0.2	−10.3 - 9.8
	Est (ES)	4.5	−0.1 - 9.8
	Haut Bassins (HB)	1.4	−3.5 - 6.8
	Nord (NO)	−0.7	−6.6 - 4.5
	Plateau Central (PC)	−7.5	−14.8 - 0.0
	Sahel (SA)	0.9	−4.3 - 6.3
	Sud Ouest (SO)	−0.7	−7.2 - 5.5
	ALL	0.4	−1.6 - 2.3

Note: the median of the temporal change does not necessarily equal the change in the median

In Ghana, the first survey round estimated that the Upper West region had the highest mCPR, whereas the Northern and Western regions had much lower rates (Fig. 2). The geographic variation remained while estimated contraceptive use rose in the country as a whole. In the last round, the use rate in upper regions (Upper West and Upper East) continued to be higher than the national average. The other visible trend is regional convergence or the reduction of subnational inequity in mCPR. The regions with low mCPR in Round 1 (R1), e.g., Volta and Eastern, have higher estimates by R4.

Change over the two-year period is less striking in Ethiopia than in Ghana, but with larger within-country variations in both level and trend. Amhara region consistently has the highest mCPR, except in round 4 when it was surpassed by Dire Dawa. mCPR in Ethiopia Somalia remains below 10% during the four rounds. Afar started round 1 with lowest regional mCPR at 5.4%; it increased to 18.2% by round 3 before dropping back to 12.9% in round 4. The mCPRs in Gambella, Harari, Oromiya, SNNPR, and Tigray have stayed in the interval of 20–30% throughout the two years.

Kenya’s subnational mCPRs are about twice the magnitude of Ghana’s and Ethiopia’s. The median change ranges from 0.6% points in Kericho County to 10.0% points in Kitui. The 95% UIs do not cross 0 in 5 of the 9 counties—Kiambu, Kitui, Nairobi, Nyamira, and Siaya, with the median change ranging from 5.2% points (Nyamira) to of 10.1% points observed in Kitui. In terms of within-country disparity, Kilifi County shows the lowest mCPR estimates, with an apparent upward trend after Round 2.

In the Central 1 region of Uganda, the median of the posterior distribution of the temporal change is 4.1 percentage points and the 95% uncertainty interval is between a decrease of 1.6 percentage points to an increase of 10.1 percentage points. It should be noted that the median is close to the difference between the point estimates for Rounds 1 and 4, but the 95% UI of the measured change is not the same as the difference between the 95% UIs for the two rounds. This is because the posterior distribution of the difference fully captures all relevant uncertainty and serial correlations.

While only two rounds of estimates are available for Burkina Faso, six of the 10 regions show positive change in mCPRs. In the other four countries, the trends in regional estimates are largely upward. In Uganda the lower bounds of 95% UIs are above 0 in 9 of 10 regions. This suggests growth in contraceptive practice at both the national and regional levels. The pattern of change in Uganda is then relatively homogenous across the country. Except for Karamoja region where the estimated mCPR registers a decrease of 0.5% points, the magnitude of variation in the mCPRs for all other 9 countries are close in value. The smallest change is observed for the Central 1 region (4.1% points) and the largest in Central 2 region (9.6% points). The 95% UIs cross 0 in only two regions (Central 1and Karamoja). The magnitudes of the estimated change are plausible considering their population-level basis.

**Fig. 3:**
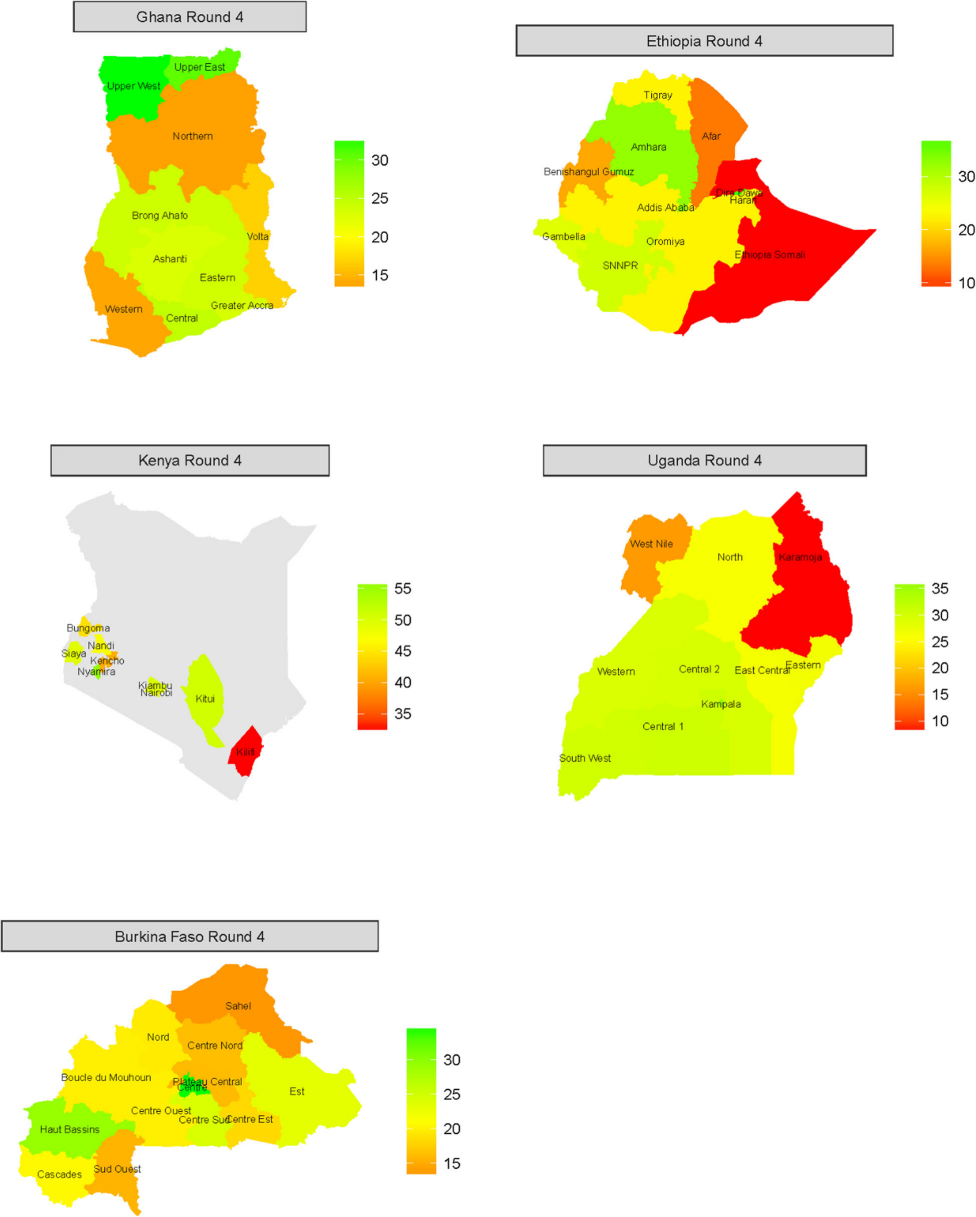
Geographic variation in Bayesian estimates of round 4 regional modern contraceptive prevalence rates for Ghana, Ethiopia, Kenya, Uganda and Burkina Faso

Figure 3 presents the R4 Bayesian model estimates for each country from Table 2 in regional maps, highlighting their geographic variation. There is noticeable geographic variation in mCPR level within each of five countries.

**Fig. 4:**
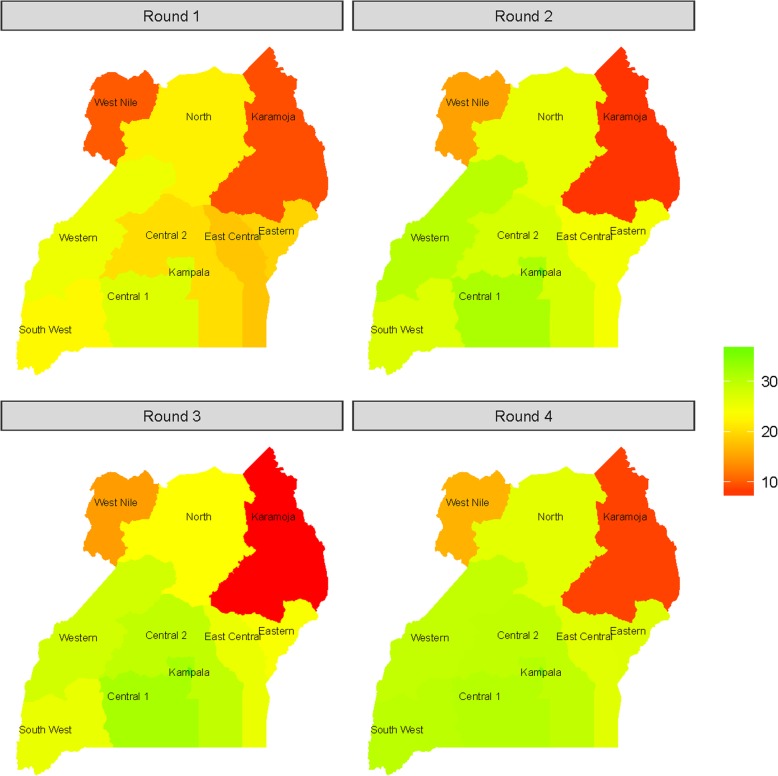
Temporal and geographic variation in Bayesian estimates over four rounds of PMA2020 survey in Uganda

Figure 4 illustrates the temporal and geographic pattern across regions for one country--Uganda. Overall, the use rate is higher in the south than the north. Karamoja and West Nile consistently has the lowest mCPR in the country. Over the four rounds of PMA2020 surveys, an upward change in mCPR is estimated to have occurred mainly in the South. Regions like South West, Western, and Central 2 started with low levels of modern contraceptive use but show higher estimates from rounds 1 to 4. There is a clear pattern, as in Ghana, of regional convergence or less disparity in levels across regions, particularly in the South. As illustrated by the assimilating colors, round 4 estimates for several regions in the South have relatively similar mCPR levels.

## Discussion

Generating subnational estimates for indicators of health and development can provide information relevant for local program officials, enabling them to pursue evidence-based priority setting and policy making. The ability to regularly monitor and evaluate programs and interventions is important for guiding performance and achieving the best possible system- and population-level health outcomes. However, in most low-income countries, there is a persisting gap between available data and evidence and information needs of local policy makers.

This study has addressed how that gap can be narrowed by maximizing the use of existing data and applying Bayesian statistical models for subnational estimates of relevant indicators, using modern contraceptive prevalence as an example. The ongoing PMA2020 surveys provide an opportunity to generate reliable subnational estimates, even though the surveys were designed primarily to provide national estimates. The detailed information collected from women and households is reasonably robust to explain and predict the use of modern contraceptive methods at subnational levels. Aligning these estimates with the administrative levels where they are needed is a key consideration in designing population and household sample surveys, particularly when conducted with the needed frequency to be useful programmatically and inevitably under resource constraints. Similarly, once the subnational estimation model is developed, having a standard for comparison is helpful. Demographic and Health Surveys (DHS) has been a frequently used source for national, in some special cases subnational, estimates of mCPR. However, because DHS data are collected only every five years, having other survey data, such as PMA2020, to generate subnational estimates of desired and consistently measured indicators more frequently will be beneficial for local program planning and monitoring community demand.

Our model results affirm the strong methodological gains of Bayesian hierarchical modeling for subnational estimation. As provided in the Additional file 1 (pp 33–37), the shrinkage plots point to acceptable improvement of subnational Bayesian over direct estimates. The examination of the standardized residual and Z-values confirm that the BHM explains and predicts modern contraceptive use well, with no apparent major violation of model assumptions (Additional file 1 pp. 27–31). The model-based estimates by subnational area for the five countries demonstrate considerable narrowing of the uncertainty interval, as compared to the direct estimates, and the temporal change in the median estimates are largely credible.

Despite our extensive and systematic effort to diagnose and improve the models, the study is not without limitations. First, the validity of our BHM relies on the selection of covariates, whose availability depends on PMA2020 questionnaire design. It is possible that our models are missing some important covariates. Second, a small proportion of women (< 5%) were interviewed in more than one round. We did not account for repeated interviews because the influence is unlikely to be substantial [28].

## Conclusion

To our knowledge, this is one of the first Bayesian hierarchical models applied to provide subnational estimates of mCPR in sub-Saharan African countries. We improved the previous global and regional models through incorporating a set of 12 woman-level covariates. From the findings, we observe substantial geographic variation in mCPRs in both levels and trends for each of the five countries. The national mCPR has been increasing in all countries, with variation across regions. Beyond the substantive findings, this study also offers a methodological contribution. As noted in the Additional file 1 (pp 27–37), the improved model diagnostic indicator, the Z-value, provides an improved option for assessing extreme values of model estimates. Given the initial semi-annual and now annual frequency of PMA2020 survey data collection and given the resource constraints of accommodating large sample size requirements for subnational estimates, applying the Bayesian estimation approach to obtain subnational indicator estimates offers important advantages and benefits for population health monitoring and program management.

## Additional file

Additional file 1: Part I. Documentation of Methods for Small Area Estimation. Part II: Detailed results. (PDF 3375 kb)

## Abbreviations

BHM: Bayesian Hierarchical Approach
DHS: Demographic and Health Surveys
EA: Enumeration Area
FP2020: Family Planning 2020
mCPR: Modern Contraceptive Prevalence Rate
PMA2020: Performance Monitoring and Accountability 2020
SDG: Sustainable Development Goals
UI: Uncertainty Interval
WRA: Women of Reproductive Ages

## References

[1] Cleland J, Bernstein S, Ezeh A, Faundes A, Glasier A, Innis J (2006). Family planning: the unfinished agenda. Lancet.

[2] Fortney JA (1987). The importance of family planning in reducing maternal mortality. Stud Fam Plan.

[3] Gwako EL (1997). Conjugal power in rural Kenya families: its influence on women's decisions about family size and family planning practices. Sex Roles.

[4] Morgan SP, Niraula BB (1995). Gender Inequality and Fertility in two Nepali villages. Popul Dev Rev.

[5] Sinha N (2005). Fertility, child work, and schooling consequences of family planning programs: evidence from an experiment in rural Bangladesh. Econ Dev Cult Chang.

[6] Chola L, McGee S, Tugendhaft A, Buchmann E, Hofman K (2015). Scaling up family planning to reduce maternal and child mortality: the potential costs and benefits of modern contraceptive use in South Africa. PLoS One.

[7] Bailey MJ, Malkova O, Norling J (2014). Do family planning programs decrease poverty? Evidence from public census data. CESifo economic studies.

[8] Assembly UNG. Transforming our world: the 2030 agenda for Sustainable Development. 2015. http://www.un.org/ga/search/view_doc.asp?symbol=A/RES/70/1&Lang=E (Accessed 6 Feb 2018.

[9] Corsi DJ, Neuman M, Finlay JE, Subramanian SV (2012). Demographic and health surveys: a profile. Int J Epidemiol.

[10] Fabic MS, Choi Y, Bird S. A systematic review of demographic and health surveys: data availability and utilization for research. Bull World Health Organ 2012; 90(8): 604–612. 10.2471/BLT.11.095513 PMC3417790 22893744

[11] Gonzalez ME, Hoza C (1978). Small-area estimation with application to unemployment and housing estimates. J Am Stat Assoc.

[12] Li W, Kelsey JL, Zhang Z (2009). Small-area estimation and prioritizing communities for obesity control in Massachusetts. Am J Public Health.

[13] Asiimwe JB, Jehopio P, Atuhaire LK, Mbonye AK (2011). Examining small area estimation techniques for public health intervention: lessons from application to under-5 mortality data in Uganda. J Public Health Policy.

[14] Rao JN, Molina I (2015). Small area estimation.

[15] Pramanik S, Muthusamy N, Gera R, Laxminarayan R (2015). Vaccination coverage in India: a small area estimation approach. Vaccine.

[16] Lin YH, McLain AC, Probst JC, Bennett KJ, Qureshi ZP, Eberth JM (2017). Health-related quality of life among adults 65 years and older in the United States, 2011-2012: a multilevel small area estimation approach. Ann Epidemiol.

[17] Kleinschmidt I, Sharp B, Mueller I, Vounatsou P (2002). Rise in malaria incidence rates in South Africa: a small-area spatial analysis of variation in time trends. Am J Epidemiol.

[18] Devine OJ, Louis TA, Halloran ME (1994). Empirical Bayes methods for stabilizing incidence rates before mapping. Epidemiology (Cambridge, Mass).

[19] New JR, Cahill N, Stover J, Gupta YP, Alkema L. Levels and trends in contraceptive prevalence, unmet need, and demand for family planning for 29 states and union territories in India: a modelling study using the Family Planning Estimation Tool. Lancet Global Health. 2017;5(3):e350–e8. 10.1016/S2214-109X(17)30033-5 28193400

[20] Alkema L, Kantorova V, Menozzi C, Biddlecom A. National, regional, and global rates and trends in contraceptive prevalence and unmet need for family planning between 1990 and 2015: a systematic and comprehensive analysis. The Lancet. 381(9878):1642–52. 10.1016/S0140-6736(12)62204-1 23489750

[21] Cahill N, Sonneveldt E, Stover J (2018). Modern contraceptive use, unmet need, and demand satisfied among women of reproductive age who are married or in a union in the focus countries of the Family Planning 2020 initiative: a systematic analysis using the Family Planning Estimation Tool. The Lancet.

[22] Mauro F, Monleon VJ, Temesgen H, Ford KR (2017). Analysis of area level and unit level models for small area estimation in forest inventories assisted with LiDAR auxiliary information. PLoS One.

[23] Hidiroglou MA, You Y (2016). Comparison of unit level and area level small area estimators. Survey Methodology.

[24] Zimmerman L, OlaOlorun F, Radloff S (2015). Accelerating and improving survey implementation with mobile technology: lessons from PMA2020 implementation in Lagos, Nigeria. Etude de la Population Africaine.

[25] Zimmerman L, Olson H, Tsui A, Radloff S (2017). PMA2020: rapid turn-around survey data to monitor family planning service and practice in ten countries. Stud Fam Plan.

[26] Carlin BP, Louis TA. Bayesian methods for data analysis: CRC Press; 2008.

[27] Uganda Bureau of Statistics - UBOS, ICF International. Uganda Demographic and Health Survey 2011. Kampala, Uganda: UBOS and ICF International. p. 2012.

[28] Hawes M, Safi S, Greenleaf A (2017). Response patterns on behavioral outcomes in relation to use of resident enumerators over multiple survey rounds.

