# Supplementary material for: Subnational estimation of modern contraceptive prevalence in five sub-Saharan African countries: a Bayesian hierarchical approach
Source: BMC Public Health. 2019 Feb 20;19:216. doi: 10.1186/s12889-019-6545-3 (PMC6383248; doi:10.1186/s12889-019-6545-3)
Supplement: Supplementary file 1 — Part I. Documentation of Methods for Small Area Estimation. Part II: Detailed results. (PDF 3375 kb) [file 12889_2019_6545_MOESM1_ESM.pdf]

Subnational estimation of modern contraceptive prevalence in five sub-Saharan African countries: a Bayesian hierarchical approach

Supplementary appendix

Part I: Documentation of Methods for Small Area Estimation

Part II: Detailed results

Part I: Documentation of Methods for Small Area Estimation

February 4, 2018

Documentation of Methods for Small Area Estimation

1 Introduction

We provide a mid-level documentation of our analytic approaches to SAE. For background and technical details on Bayesian methods see Banerjee et al. (2014); Carlin and Louis (2009); Diggle (2014); Gelman et al. (2013). See the main manuscript for details on the analysis goals, data and results.

2 Woman-level, Bernoulli Modeling

Because some covariates vary over women within an EA, modeling must be Bernoulli (0/1 outcome) at the woman-specific level, with estimates 'rolled-up' to the EA level. To fix ideas, the following is for a single survey wave and so the subscript t in Section 3 is omitted.

Notation:

- $k = 1, \ldots, K$: EA index
- $i = 1, \ldots, n_k$, indexes women in EA k.
- Y_{ik}: 0/1 indicator of woman i in region k (not using)/using birth control (or whatever other binary outcome is relevant).
- $[Y_{ik} \mid P_{ik}] \sim$ Bernoulli(P_{ik}).
- $\hat{P}_k = Y_{+k}/n_k$: direct (unadjusted) estimate for EA k.
- $\mathbf{X}_{ik}$: regressors for woman i in region k, $1 \times q$ row vector including the intercept. Note that some covariates may be EA-specific, but it's best to retain the (i, k) subscript for all covariates.
- $\boldsymbol{\beta}$: a $q \times 1$ column vector of regression slopes.
- $P_{ik} = P(\mathbf{X}_{ik}\boldsymbol{\beta} + u_k)$: the true, underlying woman-specific probability for woman i in EA k, conditional on $[\mathbf{X}_{ik}, \boldsymbol{\beta}, U_k = u_k]$.

$$\text{logit}\{P(\mathbf{X}_{ik}\boldsymbol{\beta} + u_k\} = \mathbf{X}_{ik}\boldsymbol{\beta} + u_k$$

- $U_k \sim N(0, \tau); k = 1, \ldots, K$ are the EA-specific, random effects (Note that U is indexed only by k)
  - The independence model sets $U_k \ iid \ N(0, \tau^2)$
  - A time-series model (e.g., AR1) induces between-wave correlation (see Section 3).
- The 'average logistic', Avelogistic$_k = \sum_{i=1}^{n_k} P(\mathbf{X}_{ik}\boldsymbol{\beta} + U_k)$, integrated over the posterior distribution of $\boldsymbol{\beta}$ and over the prior distribution for the U_k. The avelogistic plays the role of a standard logistic regression, but brings in uncertainty in the slopes (frequentists should do this too!), and also integrates over the *prior distribution* of the U_k.

2.1 Using the MCMC samples

Both the population parameters and EA-specific MCMC outputs are relevant to in and out of sample inferences and predictions. Most programs, including `BUGS` and `rstan`, provide some summaries of monitored features, for example their mean, median, quantiles, etc. But, by monitoring and saving all relevant values, one has access to the full joint distribution of all quantities, a distribution that includes all uncertainties.

With $\nu = 1, \ldots, M$ indexing MCMC post-burn-in, pooled over chains samples, the following are available and need to be saved. Of course, the $\mathbf{X}_k$ are also available.

- Population parameters $\left\{\boldsymbol{\beta}^{(\nu)}, \tau^{(\nu)}\right\}, \tau^{(\nu)} = \sqrt{\tau^{2\,(\nu)}}$; EA random effects ($U_k^{(\nu)}$); and the X_{ik} are combined to produce woman-specific draws from the posterior distribution:

$$P_{ik}^{(\nu)} = P\left(\mathbf{X}_{ik}\boldsymbol{\beta}^{(\nu)} + U_k^{(\nu)}\right) \quad , \nu = 1, \ldots, M; i = 1, \ldots, n_k; k = 1, \ldots, K \tag{1}$$

- These woman-specific posterior distributions are then 'rolled-up' to the EA-level, specifically, for each MCMC draw, let

$$P_{+k}^{(\nu)} = \sum_{i=1}^{n_k} P_{ik}^{(\nu)}, \nu = 1, \ldots, M; k = 1, \ldots, K \tag{2}$$

A big advantage of the MCMC approach is the availability of these samples. They can be analyzed to produce virtually any summary feature of the joint posterior distribution. For example, the posterior distribution of $P_{+1} \times P_{+}2$ is obtained by computing products using the output data and summarizing the M values.

For each k, the following, EA-specific summaries using $\left(P_{+k}^{(1)}, \ldots, P_{+k}^{(M)}\right)$ are of primary interest (of course, others can be computed):

- The full posterior distribution: the histogram or smoothed density
- The sample mean:

$$E(P_{+k} \mid \text{data}) = \bar{P}_{+k} = \frac{1}{M}\sum_{\nu}\sum_{i=1}^{n_k} P\left(\mathbf{X}_{ik}\boldsymbol{\beta}^{(\nu)} + U_k^{(\nu)}\right)$$

- SD(P_{+k} | data): the sample standard deviation of the $P_{+k}^{(\nu)}$. Note that this is the SD of the estimate, so is its SE. (Warning: using the SE for CIs is not recommended!)
- Percentile-based CI: Their 2.5th, 50th and 97.5th percentiles with the 50th being a 'point estimate' and the (2.5th, 97.5th) producing a CI. Consider using the format, ${}_{2.5}50_{97.5}$ (see Louis and Zeger, 2008)
- Moment-based CI: $\bar{P}_{+k} \pm 1.96 \times SD(P_{ik})$ (not recommended!)
  Since the posterior distribution for a P_k can be highly skewed, the percentile approach is recommended. If you want the moment-based intervals, do compute them in the logit scale (compute logits of the $P_{+k}^{(\nu)}$, do the analysis and and then invert ('expit') the endpoints. Better still, use the percentile-based CI.

- The posterior distribution of the U_k: for each EA$_k$ the 2.5th, 50th and 97.5th percentiles with the 50th being a 'point estimate' and the (2.5th, 97.5th) producing a CI.

Population parameters
Similar summaries of the population parameters are also available by data-analyzing the $\{\boldsymbol{\beta}^{(\nu)}, \tau^{(\nu)}\}, \nu = 1, \ldots, T$. In addition, you can plot, for example, β_1 versus β_2 or compute the full covariance matrix for the βs.

3 The first-order auto-regressive (AR1) model

We provide an overview; the relevant literature is needed to fill in the details. The index t denotes 'wave' and we focus on the U_{kt}. The complete model also includes the fixed-effects, $\mathbf{X}_{ikt}\boldsymbol{\beta}$ (a more general model would allow a t index on $\boldsymbol{\beta}$, so $\boldsymbol{\beta}_t$.[1] As for all regression models, the implicit assumption is that the unconditional mean structure is modeled by the fixed effects and that the U_{kt} are residuals and have marginal mean 0.

We focus on the first-order, auto-regressive (AR1) model, starting with a model that allows for a wave-specific, cross-sectional variance (τ_t^2) and then specialize to $\tau_t^2 \equiv \tau^2$. Several other time-series models are candidates for inducing longitudinal association among the U_{kt}. We outline these in Section 3.5.

3.1 The AR1 model

- U_{kt} are the EA- and wave-specific random effects, k = EA, t = wave.
- The AR1 model induces correlation, $\rho^s, \rho \in [0, 1)$ for Us that are s time units apart.[2]
  - For equally spaced time increments, $\rho^s = \text{cor}(U_{kt}, U_{k(t+s)})$
- Gaussian prior on the U_{kt} : $[U_{11}, \ldots, U_{K1} \mid \tau_t] \overset{iid}{\sim} N(0, \tau_t^2)$
- Options for the prior on the τ_t^2 (independent for each t):
  - Inverse Gamma
  - Uniform over some interval
  - In `rstan`, 'flat'
- Options for the prior for ρ
  (for AR1 and other AR models ρ should be restricted to $[0, 1]$):
  - Fisher's Z: Half-normal {restricted to $[0, \infty)$} for $Z(\rho) = 0.5 \times \log\{(1 + \rho)/(1 - \rho)\}$
  - ρ ~Uniform[0, 1.0]

3.2 AR1 conditional distributions

We present conditional distributions for a general set of τ_t^2 and when $\tau_t^2 \equiv \tau^2$.

[1] Generally, a smoothing approach would be used rather than saturating the $\boldsymbol{\beta}_t$ model.
[2] Giving $\rho < 0$ prior support is inappropriate for the AR1 model.

3.2.1 Longitudinal conditional distribution for a general τ_t^2

Here is the distribution conditional on all *previous* U-values:

$$\begin{aligned}
[U_{kt}|U_{k1},\ldots,U_{k(t-1)};\tau_1,\ldots,\tau_{t-1},\tau_t,\rho] &= [U_{kt}|U_{k(t-1)};\tau_{t-1},\tau_t,\rho] \sim N\left(\rho\tau_t\tau_{t-1}^{-1}U_{k(t-1)},(1-\rho^2)\tau_t^2\right) \\
&= \rho\tau_t\tau_{t-1}^{-1}U_{k(t-1)} + \tau_t\big(1-\rho^2\big)^{\frac{1}{2}}e_{kt} \\
e_{kt} &\sim N(0,1) \text{ independent of the Us and the other } e\text{s}
\end{aligned}$$

Notes:

1. Marginally for wave t, U_{kt} iid $N(0,\tau_t^2)$
2. Even though we condition on all of the prior Us, only the most recent is used to compute the conditional mean
3. Setting $\rho = 0$ unlinks the Us over time so that there is no 'learning' from wave to wave

3.2.2 Longitudinal conditional distribution for $\tau_t^2 \equiv \tau^2$

This is the one we'll be doing. Here is the distribution conditional on all *previous* U-values:

$$\begin{aligned}
[U_{kt}|U_{k1},\ldots,U_{k(t-1)};\tau,\rho] &= [U_{kt}|U_{k(t-1)};\tau,\rho] \sim N\left(\rho U_{k(t-1)},(1-\rho^2)\tau^2\right) \qquad (3) \\
&= \rho U_{k(t-1)} + \tau\big(1-\rho^2\big)^{\frac{1}{2}}e_{kt} \\
e_{kt} &\sim N(0,1) \text{ independent of the Us and the other } e\text{s}
\end{aligned}$$

Notes:

1. Marginally for wave t, U_{kt} iid $N(0,\tau^2)$
2. Even though we condition on all of the prior Us, only the most recent is used to compute the conditional mean
3. Setting $\rho = 0$ unlinks the Us over time so that there is no 'learning' from wave to wave

3.3 The marginal distribution when $\tau_t \equiv \tau$

Taking $K = 3$ and $\tau_t \equiv \tau$, the marginal distribution of $\mathbf{U} = (U_{k1}, U_{k2}, U_{k3})'$ with equal time-spacing is,

$$\begin{aligned}
\mathbf{U} &\sim N_3\left(\underset{\sim}{0},\tau^2\mathbf{R}\right) \\
\mathbf{R} &= \begin{pmatrix} 1 & \rho & \rho^2 \\ \rho & 1 & \rho \\ \rho^2 & \rho & 1 \end{pmatrix} \qquad (4)
\end{aligned}$$

The correlations decrease exponentially fast with time-separation. Note that as in the foregoing equations, the conditional distributions use only the most proximal Us.

3.3.1 General conditional distribution when $\tau_t \equiv \tau$

The AR1 structure isn't restricted to longitudinal relations; it depends on 'neighbors.' For example, using the covariance matrix in equation (4), we have,

$$\begin{aligned} E(U_2 \mid U_1, U_3; \tau, \rho) &= \left(\frac{2\rho}{1+\rho^2}\right)\left(\frac{U_1 + U_3}{2}\right) \\ V(U_2 \mid U_1, U_3, \tau, \rho) &= \left(\frac{1-\rho^2}{1+\rho^2}\right)\tau^2 \le (1-\rho^2)\tau^2 \end{aligned}$$

There is automatic conditioning on the two neighbors, U_1 and U_3. Doing so reduces the variance more than just conditioning on U_1 (no surprise!). More generally, with the AR1 structure

$$\begin{aligned} E(U_s \mid U_1, U_2, \ldots, U_{s-1}, U_{s+1}, \ldots; \tau, \rho) &= \left(\frac{2\rho}{1+\rho^2}\right)\left(\frac{U_{s-1} + U_{s+1}}{2}\right) \\ V(U_s \mid U_1, U_2, \ldots, U_{s-1}, U_{s+1}, \ldots; \tau, \rho) &= \left(\frac{1-\rho^2}{1+\rho^2}\right)\tau^2 \end{aligned}$$

3.4 Bringing in the fixed effects

All of the foregoing is for the residuals U_{kt}. With covariates, let $\theta_{ikt} = \text{logit}(P_{ikt})$, and for specificity $\tau_t \equiv \tau$. With $\mathbf{X}_{ik} = (\mathbf{X}_{ik1}, \mathbf{X}_{ik2}, \ldots, \mathbf{X}_{ikt})$, we have,

$$\begin{aligned} [\theta_{ikt} \mid U_{k1}, \ldots, U_{k(t-1)}; \mathbf{X}_{ik}, \boldsymbol{\beta}, \tau, \rho] &\sim N\left\{\mathbf{X}_{ikt}\boldsymbol{\beta} + \rho U_{k(t-1)}, (1-\rho^2)\tau^2\right\} \\ &= \mathbf{X}_{ikt}\boldsymbol{\beta} + \rho U_{k(t-1)} + \tau(1-\rho^2)^{\frac{1}{2}} e_{kt} \\ e_{kt} &\sim N(0,1) \text{ independent of the Us and the other } e\text{s} \end{aligned}$$

Note that the 'autoregression' is the same as equation (3), it operates on residuals.

3.5 Extensions

The AR1 model is a subset of a far more general ARIMA(p,q) (Autoregressive, Integrated Moving Average) models. The ARMA(p,q) are a subset of these, with the following representations (for a single k). The e are all iid mean 0:

$$\begin{aligned} U_t &= \sum_{\ell=1}^{p} \varphi_\ell U_{t-\ell} + e_t & \boxed{\text{ARMA(p, 0)}} \\ U_t &= e_t + \sum_{\ell=1}^{q} \theta_\ell e_{t-\ell} & \boxed{\text{ARMA(0,q)}} \\ U_t &= e_t + \sum_{\ell=1}^{p} \varphi_\ell U_{t-\ell} + \sum_{\ell=1}^{q} \theta_\ell e_{t-\ell} & \boxed{\text{ARMA(p,q)}} \end{aligned} \tag{5}$$

We don't need this degree of flexibility and also don't have a sufficient number of waves to support much more than $p + q \le 2$. We'll stay with AR1 probably forever, but it's interesting to see the relation between the ARMA(1,0) and the ARMA(0,1) covariance matrices.

Equation 4) is for the ARMA(1,0) model; the covariance for the ARMA(0,1) model, is:

$$\rho = \frac{\theta}{1+\theta^2}$$

$$\mathbf{R} = \begin{pmatrix} 1 & \rho & 0 \\ \rho & 1 & \rho \\ 0 & \rho & 1 \end{pmatrix}$$

and more generally the diagonal is 1, the first super and sub diagonals are ρ and 0 elsewhere. In this model, the correlation persist irrespective of time-separation. An ARMA(1,1) model allows for correlation that decreases with time-separation down to a positive value rather than to 0.

4 Out of Sample Prediction

We consider general predictions and also those focused only on assessing fit of the regression model. The predictive distribution captures full uncertainty in a 'future direct estimate' by including uncertainty in the predictive model and in the observed data conditional on the predictive model. The standard Bayes estimates that condition on all observeds are as specified in Section 2; none of what follows changes them.

Out of sample prediction entails using a model informed by 'training data' to generate the full predictive, possibly joint, distribution for 'out of sample' units. In our context these are a subset of EAs identified by a list of (k, t) subscripts. The following assumes that a program is available that accommodates use of 'NA' to indicate a missing direct estimate, or program the model to treat missing data items as 'parameters' and that the M post-burn-in generated imputations for the associated EA$_{kt}$ can be captured. If neither of these approaches are available, imputations need to be programmed 'by hand' (see Section ?? for a basic example). For complex models for the dependency of the U_{kt} (for example, a spatial model), it is quite challenging and most assuredly not recommended.

Note that in what follows we use Y_{kt} as shorthand for Y_{+kt}, P_{kt} for P_{+kt} etc.

4.1 The method

The high-level method is very straightforward.

Step 1: Define,

$$\mathcal{I}_{kt} = \begin{cases} 1, & \text{if EA } (k,t) \text{ is to be imputed} \\ 0, & \text{if EA } (k,t) \text{ is in the training sample} \end{cases}$$

Step 2: If $\mathcal{I}_{kt} = 1$, put 'NA' for the direct estimate, or provide the appropriate code that treats the direct estimate as a parameter.

Step 3: Run the model, and retain the M post-burn-in draws for all unknowns including the imputed 'direct estimates' for EAs with $\mathcal{I}_{kt} = 1$.

Step 4: The draws for an EA with $\mathcal{I}_{kt} = 1$ are the predictive distribution for it. In our application, denote them by $\tilde{Y}_{kt}^{(\nu)}$ and so the predicted prevalences are

$$\tilde{\hat{P}}_{kt}^{(\nu)} = \tilde{Y}_{kt}^{(\nu)}/n_{kt}, \tag{6}$$

where the tilde (˜) denotes an imputed rather than an observed value.

- The (k,t)-specific mean, $\tilde{\hat{P}}_{kt}^{(\bullet)}$ gives the point estimate prediction
- The interval with endpoints at the 2.5th and 97.5th percentiles gives the 95% *prediction interval*
- If the direct estimate, $\hat{P}_{kt}$, is available (but not used in estimating the model), then one can compute the traditional (observed - predicted)/SD standardized residuals and also the more appropriate Z-value computed from the inverse Gaussian of the percentile location, along with other diagnostics (see Section 5).

4.2 Notes

- Of course, $\mathcal{I}_{kt} \equiv 1$ for any EA for which we don't have the direct estimate. For EAs that have a direct estimate, we can choose to declare $\mathcal{I}_{kt} = 1$ to obtain the out of sample, full predictive distribution.
- There needs to be sufficient information provided by the training data (the EAs with $\mathcal{I}_{kt} = 0$) to support fitting the specified model). And, even if estimable, a model with high uncertainty posterior for the $\boldsymbol{\beta}$ or the $\boldsymbol{\tau}$ will produce broad predictions
- The model must be specified to support predictions. For example, if you want to use waves (1, 2, 3) to predict wave 4 and you want to allow for a wave-specific intercept, you need to have a way to trend the wave (1, 2, 3) intercepts to wave 4. The base case of 'no change' is a single column of 1s in the X-matrix $(\mu_1 = \mu_2 = \mu_3 = \mu_4)$. A linear trend is produced by two columns in the X-matrix, a column of 1s and a column $(0,1,2,3)'$, producing $\mu_t = \beta_0 + \beta_1(t-1)$, etc. Wave-specific intercepts are produced by using the full 4 degrees of freedom with the most directly interpretable being suppressing the overall intercept and including 4 columns in the design matrix with the tth column having a 1 in the tth location and 0s elsewhere.

5 Diagnostics

The full predictive distribution supports a wide variety of additional fit and performance assessments. If the modeling is correct or at least reasonably so, then the distribution of the ensemble, $\left\{\tilde{\hat{P}}_{kt}^{(\nu)}\right\}, \nu = 1, \ldots, M$ is an accurate depiction of location, spread, shape, etc. of the full predictive distribution. If not, then the direct estimates, $\hat{P}_{kt}$ will not come from their respective, computed predictive distributions. One measure of this departure is that the collection of percentile locations will depart from U(0,1) and so also the inverse Gaussian transform will depart from a N(0,1) distribution. For model diagnostics, the following should only be used for (k,t) pair with $\mathcal{I}_{kt} = 1$.

5.1 Prediction mean, variance and SD

Mean: The prediction mean is,

$$E_{kt} = \tilde{\hat{P}}_{kt}^{(\bullet)} = \frac{1}{M}\sum_{\nu=1}^{M} \tilde{\hat{P}}_{kt}^{(\nu)} \quad \text{(the predicted prevalence)} \tag{7}$$

Note 1: **For EAs with $\mathcal{I}_{kt} = 1$:** $E_{kt} = \hat{\tilde{P}}_{kt}^{(\bullet)}$ is the general version of '**Avelogistic**$_{kt}$,' the predicted value that conditions on information **other than** the direct estimate. Therefore, this general definition of Avelogistic is the appropriate X-axis in assessing fit of the (logistic) regression model coupled with the assumed model for association amongst the U-values and the τ_t. The full predictive distribution is appropriate evaluating for a wide variety of out of sample predictions, for example wave 4 direct estimates, using wave (1,2,3) training data along with the Xs for wave 4 and a joint distribution assumption on the Us (e.g, AR1). Performance can be compared for different sets of Xs, different assumptions on relations among the Us, and among the τ_t.

Note 2: Avlogistic$_{kt}$ for an EA with $\mathcal{I}_{kt} = 1$ mixes over the $[U_{kt} \mid U_{\ell s}, \ell \neq k, s \neq t]$.

(a) For example, if the model being fit specifies that the U_{kt} are completely independent, then Avlogistic$_{kt}$ for an EA with $\mathcal{I}_{kt} = 1$ mixes over the prior distribution for that U_{kt}.

(b) If the model being fit specifies association among the Us (e.g., is spatial or autoregressive), then the posterior distribution 'learns' from other U-values.

Note 3: **For EAs with $\mathcal{I}_{kt} = 0$:** $E_{kt} = \hat{\tilde{P}}_{kt}^{(\bullet)}$ is the posterior mean that, in addition to other conditioning, conditions on the EA-specific direct estimate. The collection, $\left\{\hat{\tilde{P}}_{kt}^{(\nu)}\right\}, \nu = 1, \ldots, M$ provide the full, posterior distribution. E_{kt} should not be used as the X-axis in a residual plot, but is the Bayes posterior mean estimate for EA k in wave t, and is the standard point estimate for comparing EAs, coloring maps, etc. The full distribution should be used for CIs (in Bayes-speak 'credible intervals') and the lengths of these to color maps, etc.

Note 4: The full predictive distribution supports point estimates other than the predictive mean. For example, in some applications the predictive median is more appropriate and in this case 'mean' should be replaced by 'median' in the foregoing Notes. More generally, pick your favorite one number summary (e.g., the 10% trimmed mean) and use it!

5.2 Mean, variance, SD of a residual

Equations (7) and (8) are used to compute the residual and the standardized residual, should only be used for the (k,t) pairs with $\mathcal{I}_{kt} = 1$, and of course they depend on availability of the direct estimate. The sample variance of the $\hat{\tilde{P}}_{kt}^{(\nu)}$ is:

$$\begin{aligned} V_{kt} &= \frac{1}{M}\sum_{\nu=1}^{M}\left(\hat{\tilde{P}}_{kt}^{(\nu)} - E_{kt}\right)^2 \\ \mathsf{SD}_{kt} &= V_{kt}^{\frac{1}{2}} \end{aligned} \tag{8}$$

The direct estimate and residuals are:

$$\begin{aligned} \hat{P}_{kt} &= \frac{Y_{+kt}}{n_{kt}} \quad \text{(the direct estimate)} \\ \hat{R}_{kt} &= (\hat{P}_{kt} - E_{kt}) \\ R^*_{kt} &= \frac{\hat{R}_{kt}}{\mathsf{SD}_{kt}} = \frac{\hat{P}_{kt} - E_{kt}}{\mathsf{SD}_{kt}} \end{aligned} \tag{9}$$

Residuals $\hat{R}_{kt}$ and R^*_{kt} in equation (9) are based on the observed direct estimate $(\hat{P}_{kt})$ and so measure discrepancy from the assumed model with R^*_{kt} calibrated by the standard deviation of the predictive distribution. If the direct estimates have close to a Gaussian distribution, then the R^*_{kt} can be used to make residual plots, histograms, boxplots, QQ plots, etc. However, if the direct estimates are not close to Gaussian, then use the percentile approach described in Section 5.3. In any case, don't use the $\hat{R}_{kt}$ for any diagnostics, because they haven't been calibrated by their standard deviation.

5.3 Using the full predictive distribution

The formulas in display (9) measure deviation in standard deviation units, but other measures less closely tied to the Gaussian distribution are available. The following are effective diagnostics, but any computation using the ensemble that targets fit is 'legal.' The following should only be computed for (k, t) with $\mathcal{I}_{kt} = 1$!

1. Find the **percentile location** of $\hat{P}_{kt}$ amongst the $\left\{\tilde{\hat{P}}^{(\nu)}_{kt}\right\}$, denote it by ζ_{kt}, and use for the standardized residual,

$$R^{\ddagger}_{kt} = \begin{cases} -4.0, & \text{if } \hat{P}_{kt} \text{ is below the range of the predictive distribution} \\ \Phi^{-1}(\zeta_{kt}), & \text{if } \hat{P}_{kt} \text{ is in the range of the predictive distribution} \\ 4.0, & \text{if } \hat{P}_{kt} \text{ is above the range of the predictive distribution} \end{cases}$$

See Cook et al. (2006) for a similar approach and Efron (2008) for an example of transforming to z-values.

To compute the percentile location it's important to move away from 0 and 1 and to account for ties. So, do the following,

$$\begin{aligned} \zeta_{kt} &= \frac{\#\left\{\tilde{\hat{P}}^{(\nu)}_{kt} < \hat{P}_{kt}\right\} + \frac{1}{2}\#\left\{\tilde{\hat{P}}^{(\nu)}_{kt} = \hat{P}_{kt}\right\}}{M} \\ &= \frac{2 \times \#\left\{\tilde{\hat{P}}^{(\nu)}_{kt} < \hat{P}_{kt}\right\} + \#\left\{\tilde{\hat{P}}^{(\nu)}_{kt} = \hat{P}_{kt}\right\}}{2M} \end{aligned} \tag{10}$$

Note that this ratio is strictly greater than 0 and strictly less than 1. Also, if all of the MCMC draws equal $\hat{P}_{kt}$, then $\zeta = \frac{1}{2}$ and the residual is 0, as it should be.

If the predictive distribution is exactly Gaussian, these will be identical to the R^*_{kt} and in general are less dependent on the Gaussian assumption.

For example, if the predictive distribution were a single binomial (not our case!), here are comparisons of R^* and $R^{\ddagger}$ when the direct estimate is 0. The formulas are:

$$\begin{aligned} R^* &= -\left(\frac{np}{1-p}\right)^{\frac{1}{2}} \\ R^{\ddagger} &= \Phi^{-1}\left\{0.5 \times (1-p)^n\right\} \end{aligned}$$

Of special note is that for small values of p, $R^{\ddagger} > R^*$, and as p increases the relation reverses for $n = 25$ (Table 1), but not for $n = 5$ (Table 2). Similar relations hold for smaller values of n. The $R^{\ddagger}$ residuals are more appropriate in that they pay attention to the details of the distribution. This benefit also applies when the approach is applied to the full, predictive distribution when producing residuals Q-Q plots, etc.

p	0.005	.01	.05	.10	.50
R^*	-0.35	-0.50	-1.15	-1.67	-5.00
$R^\ddagger$	-0.15	-0.28	-1.09	-1.80	-5.54

Table 1: Residuals when the predictive distribution is Bernoulli(n = 25, p) and the direct estimate is 0. This is not our exact situation because our full predictive distribution is composed of a sum of not necessarily identically distributed Bernoulli variates and it also includes uncertainty in the probability (uncertainty in p).

p	0.005	.01	.05	.10	.50
R^*	-0.16	-0.22	-0.51	-0.75	-2.24
$R^\ddagger$	-0.03	-0.06	-0.29	-0.54	-2.15

Table 2: Residuals when the predictive distribution is Bernoulli(n = 5, p) and the direct estimate is 0. This is not our exact situation because our full predictive distribution is composed of a sum of not necessarily identically distributed Bernoulli variates and it also includes uncertainty in the probability (uncertainty in p).

2. **Box-plots:** and other outlier diagnostics using the R^*_k or $R^\ddagger_k$.
3. **Residual plots:** with either R^*_k or $R^\ddagger_k$ on the Y-axis and the appropriate Avelogistic$_{kt}$ on the X-axis. Importantly, these X-axis values should be for an MCMC run with $\mathcal{I}_{kt} = 1$ (see Note 1 below equation 7).
4. **Q-Q plots:** of the R^*_{kt} or the $R^\ddagger_{kt}$ against a Gaussian (normal) reference. This will be a good diagnostic, but because $\hat{P}_{kt}$ and the predictive distributions are computed, in part, from sums of 0/1 variables, even under the null hypothesis the distribution won't be exactly N(0,1).
   i. Equivalently, a Q-Q plot of the one-sided P-values computed using the R^*_{kt} or directly using the ζ_{kt}, against a U(0,1) reference.
5. **Chi-square goodness of fit:** When using (Observed - Predicted)/SD),

$$\chi^2_{df} = \sum_1^K (R^*_{kt})^2 \quad \text{(see equation 9)} \tag{11}$$

or, when using the percentile approach

$$\chi^2_{df} = \sum_1^K \left(R^\ddagger_{kt}\right)^2 \quad \text{(see Diag 1)}$$

The exact df needs to be determined and will depend on whether the assessment is in or out of sample, and on the correlation structure assumed for the U_{kt}. It is surely no greater than K and for the AR1 or a spatial model considerably smaller.

5.4 Additional summaries

Out of sample residuals are central to assessing the performance of a model, but shouldn't be the only components a report or an evaluation. Here are a few others, with not intention to provide a complete list.

5.4.1 Shrinkage plots

In addition to the residual plot in Section 5.3, two 'shrinkage plots' are informative.

Direct estimate to Bayes:

- Direct estimates plotted horizontally sufficiently far above the X-axis
- Whisker for each proportional to the length of the 95%, Binomial likelihood-based CI using the numerator and denominator of the direct estimate
- Bayes estimates plotted horizontally on the X-axis
- Lines connecting the Direct and the Bayes

(Direct - Avelogistic) to (Bayes - Avelogistic):

- (Direct - Avelogistic) plotted horizontally sufficiently far above the X-axis
- Whisker for each proportional to the length of the 95%, Binomial likelihood-based CI using the numerator and denominator of the direct estimate
- (Bayes - Avelogistic) plotted horizontally on the X-axis
- Lines connecting the Direct and the Bayes
  - **Note:** Unlike in Gaussian/Gaussian model, the signs of (Direct - Avelogistic) and (Bayes - Avelogistic) can differ; a line can cross 0. Crossing can occur when Avelogistic is sufficiently far from 0.5 and the Direct estimate is sufficiently close to Avelogistic.

5.4.2 The degree of shrinkage

The degree of shrinkage for EA_{kt} is,

$$\text{Shrinkage}_{kt} = \frac{\text{Direct}_{kt} - \text{Bayes}_{kt}}{\text{Direct}_{kt} - \text{Avelogistic}_{kt}} \tag{12}$$

The between-EA variance (τ^2) plays a role in how much an estimate shrinks towards the regression model; itÕs all relative. For example, if τ^2 is small relative to the variance of a direct estimate (more properly, if the posterior distribution of τ^2 has most of its mass far below the variance of the direct estimate), then the regression model will get a lot of weight even if the variance of the direct estimate is small. On the other hand, if the posterior distribution of τ^2 has most of its mass far above the variance of the direct estimate, then the regression model will get relatively little weight.

5.4.3 *Reduction in uncertainty*

Because the SD isn't the best summary for binomial and other non-Gaussian data, it is far better to compare the length of the PROPERLY COMPUTED, EXACT 95% CI associated with the direct estimate[3] and the length of the 95% probability content of the Bayes credibility interval (provided by the MCMC output). Their ratio gives a good indication of the improved stability conferred by the Bayesian model. If the lengths of the CIs based on the direct estimates and the Bayes estimates are similar, then there has been no 'Bayes advantage' and unless the direct estimates are all very stable (in which case there is no reason the stabilize them), it is worth looking for additional covariates (or transforms of current, interactions of current) that have predictive power and thereby shift the posterior distribution of τ^2 closer to

[3] In R use binconf with method = 'exact'

0. If there are no such covariates, then so be it, we need to live with what we have. I stress comparisons should be on CI length, because while for Gaussian data itÕs equivalent to comparing SDs, for count data, especially when an estimate is near 0, they aren't equivalent.

Related, as indicated in Section 5.4.1 use exact 95% CI length for whiskers: For our shrinkage plots it is better to set the whiskers proportional to the length of the exact 95% CI associated with the direct estimate rather than proportional to the SD.

5.4.4 Model criticism

As in evaluating a standard (non-Bayesian) regression or logistic regression, we donÕt expect that the residuals will all be very close to 0, but for a good model we do expect that the standardized residuals will look reasonable relative to random variables that have mean 0 and variance 1 (not necessarily Gaussian). Ditto for the Bayesian approach and with the percentile method for residuals the Zs should be close to Gaussian.

As for standard diagnostics, patterns matter as much as magnitude, and plotting standardized residuals (ideally the percentile-based ones) versus the relevant Avelogistic values is a good way to identify model lack of fit that might be reduced by including additional covariates including carefully chosen interactions based on currently included covariates. The issue here are essentially identical to traditional modeling.

Traditional models use AIC, BIC, adjusted R^2, and other one-number summaries to assess fit. In addition, Bayesian models with MCMC support DIC which is interpreted in a manner similar to AIC and BIC.

6 Aggregation Diagnostics

Comparing aggregated posterior mean or median estimates to the direct estimate associated with the aggregated regions can help diagnose model inadequacy. Aggregation needs to be sufficient so that the aggregated direct estimates and the aggregated Bayes estimates are stable and can be trusted. Subject to this requirement, any aggregation is 'fair game' with the most spatially logical being to aggregate nested domains (e.g., EAs aggregated to regions, regions to the country). These comparisons can be 'decorated' with uncertainty estimates (see Section 5), but with sufficient aggregation, uncertainty will be relatively small.

6.1 Country-level aggregation

Recall that we use the shorthand $Y_{kt} = \sum_{i=i}^{n_{kt}} Y_{ikt}$, etc. Aggregating EAs to the country-level is straightforward. For a fit assessment, compute a weighted average of the $Y_{kt}^{(\nu)}$, producing,

$$Y_{\mathbf{w}t}^{(\nu)} = \sum_k w_{kt} Y_{kt}^{(\nu)}, \nu = 1, \ldots, M \tag{13}$$

and see where $\hat{P}_{\mathbf{w}t} = \sum_k w_{kt}\hat{P}_{kt}$ falls in the distribution. The w_{kt} need to be specified; use $w_{kt} = n_{kt}/n_{+t}$, if the n_{kt} are proportional to the population size (i.e., the number of eligible women) of region k; otherwise use weights based on the true population sizes. Section 6.2 provides additional details. This computation is equivalent to comparing $\hat{P}_{\mathbf{w}t}$ to the aggregated $E_{\mathbf{w}t} = \sum_k w_{kt}E_{kt}$. It produces a single number, but can be helpful.

6.2 General aggregation

Let $\mathcal{A}$ indicate the EAs to be aggregated. That is, $\mathcal{A}$ is a list of subscripts $\{k_1, k_2, \ldots, k_{|\mathcal{A}|}\}$, where $|\mathcal{A}|$ is the number of subscripts in $\mathcal{A}$. Then, compute,

$$Y^{(\nu)}_{\mathbf{w}t|\mathcal{A}} \quad = \quad \left(\frac{1}{\sum_{k \in \mathcal{A}} w_{kt}}\right) \sum_{k \in \mathcal{A}} w_{kt} Y^{(\nu)}_{kt}, \nu = 1, \ldots, M \tag{14}$$

and use as in Section 6.1 Compute the foregoing for a collection of $\mathcal{A}$ that partition the EA space (e.g., regions), look at patterns, etc. Any partition can be used, ideally motivated by substantive considerations such as aggregated urban and aggregated rural. The collection of these aggregations can be very helpful in diagnosing model inadequacies, especially when aggregation is sufficient so that the aggregated direct are stable.

6.3 Benchmarking

The goal is to 'roll up' to a target prevalence, for example the directly estimated country prevalence. If the modeling is reasonably good, the rolled-up (usually weighted by EA sample size) Bayesian estimates should come close to the target. If not, either it's an inappropriate target or the modeling wasn't very good. In any case, estimates can be adjusted (benchmarked) to produce the match. There are a variety of ways to force the Bayes estimates (the posterior means) to benchmark, and this is the subject of a forthcoming section. Suffice for now that there are two views on forcing a benchmark:

- Force benchmarking so that the estimates are 'face-valid' to stakeholders.
- Don't force benchmarking; notable discrepancies indicate model inadequacy and these should be remedied.

The most naive, and definitely not recommended, is to *rake* the estimates by applying a common factor to the EA-specific estimates (Bayes or otherwise). For example, if the rolled-up Bayes estimates are 1% higher than the target, divide each of them by 1.01 to guarantee the match. More appropriate is to optimize predictions, subject to a (linear) benchmarking constraint (see, Bell et al., 2013), replacing,

> 'EA-specific estimates are the mean of the posterior distribution; they minimize posterior squared-error loss.'

By,

> 'EA-specific estimates minimize posterior-squared error loss, *subject to the linear constraint that they roll up to the target*.'

Though the foregoing is very appropriate for benchmarking posterior means, it can't deal with non-standard goals such as ranks and it's not clear what benchmarking would mean in that context. However, recent work I'm doing with Beka Steorts (Duke), embeds the benchmarking in the full posterior distribution so that any quantity computed from it will be 'benchmarked.' Work on this idea continues.

Bibliography

Banerjee, S., Carlin, B. P., and Gelfand, A. E. *Hierarchical Modeling and Analysis of Spatial Data (Second Edition)*. Chapman and Hall/CRC (2014).

Bell, W. R., Datta, G. S., and Ghosh, M. "Benchmarking Small Area Estimators." *Biometrika*, 100:189–202 (2013).

Carlin, B. P. and Louis, T. A. *Bayesian Methods for Data Analysis, 3rd edition*. Boca Raton, FL: Chapman and Hall/CRC Press, 3nd edition (2009).

Cook, S. R., Gelman, A., and Rubin, D. B. "Validation of software for Bayesian models using posterior quantiles." *J. Comput. Graph. Statist.*, 15:675–692 (2006).

Diggle, P. J. *Statistical Analysis of Spatial and Spatio-Temporal Point Patterns*. Chapman and Hall/CRC (2014).

Efron, B. "Microarrays, Empirical Bayes and the Two-Group Model." *Statistical Science*, 23:1–22 (2008).

Gelman, A., Carlin, J., Stern, H. S., Dunson, D. B., Vehtari, A., and Rubin, D. *Bayesian Data Analysis, 3rd edition*. Boca Raton, FL: Chapman and Hall/CRC Press (2013).

Little, R. J. "To Model or Not To Model? Competing Modes of Inference for Finite Population Sampling." *Journal of The American Statistical Association*, 99:546–556 (2004).

—. "Calibrated Bayes, an Alternative Inferential Paradigm for Official Statistics (with discussion)." *Journal of Official Statistics*, 28:309–372 (2012).

Louis, T. A. and Zeger, S. L. "Effective communication of standard errors and confidence intervals." *Biostatistics*, 10:1–2 (2008).

Part II: Detailed Results

PMA2020 sample design

PMA2020 survey samples are designed to provide estimates of the mCPR indicator with a margin of error of 2 percentage points at the national level and within 3 percentage points for rural and urban areas separately. Burkina Faso, Ghana and Uganda surveys employed a two-stage sampling strategy (urban-rural strata and then enumeration area (EA)). Ethiopia and Kenya had an additional level, stratifying first by region and county respectively. Once stratified by rural/urban residence, EAs were randomly selected in all countries. In selected EAs, all households were mapped and listed, and then with a random start, between 35 and 42 households were systematically selected for interviews. The EA size varied depending on the expected response rate and number of eligible women per household.

Local government agencies that sponsored the PMA2020 surveys often requested the sample be designed to provide subnational estimates for their administrative divisions. This has challenged the project's limited resources, usually leading to some but not all subnational units being accommodated in the sampling. Specifically, in addition to the stratification by urban-rural residency, Ethiopia and Kenya had an additional level, stratifying first by region and county respectively. In Ethiopia, of the 11 regions five account for more than 80% of the country's populations and were identified for subnational sampling, while the remaining six combined into a residual region group. EAs were allocated proportionally across the six regional groups. In Kenya, following the 2013 general election, 47 counties constituted the government's Level-1 administrative units. The Kenyan National Council for Population and Development and the Ministry of Health sought county-level estimates from the PMA2020 surveys. With a probability proportional to size (PPS) approach and within allowable resources, nine counties were selected to provide county-level estimates while in the aggregate also providing national and urban-rural estimates of mCPR. These nine counties encompass almost 30 percent of the population based on the 2009 census. Over time, independently drawn EAs were added to the samples for Ethiopia and Kenya. However, the analytic samples for this study are based on the EA samples consistently included across all four rounds (or two rounds in Burkina Faso).

Table 1: Woman-level outcome, covariates and their definitions

Indicator	Definitions of indicators and categories
**<u>Outcome</u>**	
**Modern contraceptive use**	Whether currently using a modern contraceptive method
**<u>Covariates</u>**	
**Residence**	Urban, rural, metropolitan residence
**Schooling**	Highest level attained: No education, primary, secondary or above
**Wealth quintile**	Five groups of approximately equal size based on a factor analysis score constructed from household assets
**Child survival**	Whether the last child born in the preceding two years is still alive
**Age**	Five-year age groups (15-19 years, …, 45-49 years)
**Cohabitation**	Not married; married and living with husband; married but not living with husband
**Recent sex**	Whether had sex in the past 4 weeks
**Health worker visit**	Whether visited at home by a health worker in the last 12 months
**FP message**	Whether heard a family planning (FP) message on radio/TV or saw in print in past 12 months
**Fertility intention**	Whether desires her next pregnancy 24 months or later
**Parity**	Number of live births
**Distance**	Distance (km, log transformed) to the nearest facility providing three or more modern contraceptive methods

Table 2a: Sample Characteristics for Ghana PMA Rounds 1-4

Indicator	Round									
	1		2		3		4		Total	
	No.	%	No.	%	No.	%	No.	%	No.	%
**Modern contraceptive use**										
Yes	591	14.0	520	14.4	770	18.1	1,108	22.8	2,988	17.6
No	3,636	86.0	3,102	85.6	3,476	81.9	3,746	77.2	13,960	82.4
**Residence**										
Rural	1,983	46.9	1,677	46.3	1,606	37.8	1,769	36.5	7,036	41.5
Urban	1,457	34.5	1,315	36.3	1,834	43.2	2,228	45.9	6,835	40.3
Metropolitan	786	18.6	629	17.4	806	19.0	856	17.6	3,077	18.2
**Schooling**										
No education	893	21.1	753	20.8	790	18.6	871	18.0	3,307	19.5
Primary school	770	18.2	686	18.9	733	17.3	872	18.0	3,061	18.1
Secondary school	2,564	60.7	2,183	60.3	2,723	64.1	3,110	64.1	10,580	62.4
**Wealth quintile**										
Poorest	784	18.6	826	22.8	844	19.9	1,053	21.7	3,507	20.7
Poorer	793	18.8	656	18.1	808	19.0	956	19.7	3,213	19.0
Middle	771	18.2	734	20.3	928	21.9	982	20.2	3,414	20.1
Richer	869	20.6	717	19.8	866	20.4	917	18.9	3,370	19.9
Richest	1,010	23.9	688	19.0	800	18.8	945	19.5	3,444	20.3
**Last child died**										
Yes	53	1.3	27	0.7	30	0.7	37	0.8	147	0.9
No	4,174	98.7	3,595	99.3	4,216	99.3	4,816	99.2	16,801	99.1
**Age group**										
15-19 years	767	18.2	685	18.9	777	18.3	977	20.1	3,207	18.9
20-24 years	772	18.3	621	17.1	796	18.7	926	19.1	3,114	18.4
25-29 years	779	18.4	642	17.7	746	17.6	831	17.1	2,998	17.7
30-34 years	578	13.7	567	15.7	658	15.5	749	15.4	2,553	15.1
35-39 years	561	13.3	490	13.5	526	12.4	591	12.2	2,168	12.8
40-44 years	429	10.1	329	9.1	370	8.7	389	8.0	1,516	8.9
45-49 years	341	8.1	288	7.9	373	8.8	391	8.0	1,392	8.2
**Cohabitation**										
Not married	1,433	33.9	1,458	40.2	1,810	42.6	2,124	43.8	6,825	40.3
Live together	1,872	44.3	1,573	43.4	1,726	40.6	1,918	39.5	7,089	41.8
Not living together	921	21.8	591	16.3	710	16.7	811	16.7	3,033	17.9
**Had sex last 4 weeks**										
Yes	1,785	42.2	1,545	42.7	1,916	45.1	2,462	50.7	7,708	45.5
No	2,442	57.8	2,076	57.3	2,330	54.9	2,391	49.3	9,239	54.5
**Visited by health worker**										
Yes	717	17.0	571	15.8	573	13.5	613	12.6	2,475	14.6
No	3,509	83.0	3,051	84.2	3,673	86.5	4,240	87.4	14,473	85.4
**FP message**										
Yes	3,176	75.1	2,608	72.0	3,140	74.0	3,697	76.2	12,622	74.5
No	1,051	24.9	1,013	28.0	1,106	26.0	1,156	23.8	4,326	25.5
**Desire to postpone**										
Yes	1,439	34.0	1,179	32.6	1,358	32.0	1,695	34.9	5,671	33.5
No	2,788	66.0	2,442	67.4	2,888	68.0	3,159	65.1	11,277	66.5
	Mean /SD	Median	Mean /SD	Median	Mean /SD	Median	Mean /SD	Median	Mean /SD	Median
**Parity**	2.3/2.3	1.7	2.2/2.3	1.6	2.0/2.1	1.5	1.9/2.1	1.3	2.1/2.2	1.5
**Distance**	7.3/9.6	2.2	8.0/11.5	2.2	3.4/6.8	0.8	3.1/6.0	0.74779	5.2/8.8	1.1
**Total**	4,227	100.0	3,621	100.0	4,246	100.0	4,853	100.0	16,948	100.0
Note: Sample of females 15 to 49 years of age. See Table 1 for variable definitions.										

Table 2b: Sample Characteristics for Ethiopia PMA Rounds 1-4

Indicator	Round									
	1		2		3		4		Total	
	No.	%	No.	%	No.	%	No.	%	No.	%
**Modern Contraceptive**										
Yes	1,231	23.4	1,484	24.4	1,580	26.8	1,596	27.0	5,890	25.4
No	4,023	76.6	4,599	75.6	4,324	73.2	4,313	73.0	17,259	74.6
**Residence**										
Rural	3,990	75.9	4,598	75.6	4,344	73.6	4,308	72.9	17,240	74.5
Urban	872	16.6	1,165	19.2	1,212	20.5	1,267	21.4	4,516	19.5
Metropolitan	392	7.5	319	5.3	348	5.9	334	5.7	1,394	6.0
**Schooling**										
No education	2,284	43.5	2,604	42.8	2,490	42.2	2,300	38.9	9,678	41.8
Primary school	2,020	38.4	2,312	38.0	2,207	37.4	2,315	39.2	8,854	38.2
Secondary school	950	18.1	1,166	19.2	1,207	20.4	1,295	21.9	4,618	19.9
**Wealth quintile**										
Poorest	850	16.2	1,022	16.8	1,048	17.8	1,043	17.6	3,963	17.1
Poorer	877	16.7	1,146	18.8	1,075	18.2	1,038	17.6	4,137	17.9
Middle	943	17.9	1,170	19.2	1,115	18.9	1,072	18.1	4,300	18.6
Richer	1,163	22.1	1,288	21.2	1,196	20.3	1,234	20.9	4,882	21.1
Richest	1,420	27.0	1,456	23.9	1,470	24.9	1,522	25.8	5,868	25.4
**Last child died**										
Yes	76	1.5	114	1.9	104	1.8	138	2.3	433	1.9
No	5,177	98.5	5,969	98.1	5,799	98.2	5,771	97.7	22,716	98.1
**Age group**										
15-19 years	1,197	22.8	1,426	23.4	1,389	23.5	1,401	23.7	5,413	23.4
20-24 years	901	17.2	1,114	18.3	1,105	18.7	1,032	17.5	4,152	17.9
25-29 years	983	18.7	1,226	20.2	1,041	17.6	1,058	17.9	4,308	18.6
30-34 years	750	14.3	791	13.0	774	13.1	814	13.8	3,129	13.5
35-39 years	714	13.6	753	12.4	766	13.0	694	11.7	2,927	12.6
40-44 years	412	7.8	447	7.4	451	7.6	501	8.5	1,811	7.8
45-49 years	295	5.6	326	5.4	378	6.4	410	6.9	1,410	6.1
**Cohabitation**										
Not married	1,688	32.1	2,152	35.4	2,178	36.9	2,270	38.4	8,288	35.8
Live together	3,050	58.1	3,705	60.9	3,470	58.8	3,416	57.8	13,641	58.9
Not live together	515	9.8	226	3.7	256	4.3	223	3.8	1,220	5.3
**Had sex last 4 weeks**										
Yes	2,624	49.9	3,257	53.5	3,418	57.9	3,326	56.3	12,625	54.5
No	2,630	50.1	2,826	46.5	2,485	42.1	2,583	43.7	10,525	45.5
**Visited by health worker**										
Yes	1,142	21.7	1,105	18.2	1,224	20.7	1,023	17.3	4,495	19.4
No	4,111	78.3	4,978	81.8	4,680	79.3	4,886	82.7	18,654	80.6
**FP message**										
Yes	2,158	41.1	2,506	41.2	2,562	43.4	2,592	43.9	9,819	42.4
No	3,095	58.9	3,576	58.8	3,342	56.6	3,317	56.1	13,330	57.6
**Desire to postpone**										
Yes	1,391	26.5	1,874	30.8	1,992	33.7	2,116	35.8	7,373	31.8
No	3,862	73.5	4,209	69.2	3,912	66.3	3,792	64.2	15,776	68.2
	Mean/SD	Median	Mean/SD	Median	Mean/SD	Median	Mean/SD	Median	Mean/SD	Median
**Parity**	2.2/2.5	1.4	2.1/2.5	1.2	2.1/2.5	1.2	2.1/2.5	1.2	2.1/2.5	1.3
**Distance**	4.6/10.2	1.0	1.6/3.3	0.8	1.6/3.0	0.8	1.6/3.4	0.8	2.3/5.9	0.8
**Total**	5,253	100.0	6,083	100.0	5,904	100.0	5,909	100.0	23,149	100.0
Note: Sample of females 15 to 49 years of age. See Table 1 for variable definitions.										

Table 2c: Sample Characteristics for Kenya PMA Rounds 1-4

Indicator	Round									
	1		2		3		4		Total	
	No.	%	No.	%	No.	%	No.	%	No.	%
**Modern contraceptive use**										
Yes	1,534	41.7	1,723	40.3	2,010	46.5	2,207	46.0	7,475	43.7
No	2,147	58.3	2,556	59.7	2,313	53.5	2,596	54.0	9,612	56.3
**Residence**										
Rural	2,104	57.1	2,590	60.5	2,616	60.5	2,947	61.4	10,256	60.0
Urban	866	23.5	920	21.5	969	22.4	1,065	22.2	3,821	22.4
Metropolitan	711	19.3	769	18.0	738	17.1	791	16.5	3,010	17.6
**Schooling**										
No education	142	3.9	158	3.7	187	4.3	187	3.9	675	4.0
Primary school	1,830	49.7	2,151	50.3	2,107	48.7	2,341	48.7	8,429	49.3
Secondary school	1,709	46.4	1,970	46.0	2,029	46.9	2,274	47.4	7,982	46.7
**Wealth quintile**										
Poorest	625	17.0	858	20.0	893	20.7	1,023	21.3	3,398	19.9
Poorer	635	17.2	892	20.8	890	20.6	1,014	21.1	3,431	20.1
Middle	662	18.0	833	19.5	879	20.3	981	20.4	3,354	19.6
Richer	810	22.0	834	19.5	797	18.4	840	17.5	3,280	19.2
Richest	950	25.8	863	20.2	865	20.0	945	19.7	3,623	21.2
**Last child died**										
Yes	45	1.2	31	0.7	55	1.3	44	0.9	175	1.0
No	3,636	98.8	4,248	99.3	4,269	98.7	4,758	99.1	16,911	99.0
**Age group**										
15-19 years	487	13.2	813	19.0	684	15.8	967	20.1	2,950	17.3
20-24 years	812	22.0	803	18.8	928	21.5	974	20.3	3,517	20.6
25-29 years	861	23.4	860	20.1	906	21.0	968	20.2	3,595	21.0
30-34 years	560	15.2	576	13.5	631	14.6	656	13.6	2,423	14.2
35-39 years	439	11.9	501	11.7	481	11.1	553	11.5	1,974	11.6
40-44 years	298	8.1	446	10.4	357	8.3	376	7.8	1,477	8.6
45-49 years	226	6.1	280	6.6	335	7.8	308	6.4	1,149	6.7
**Cohabitation**										
Not married	1,280	34.8	1,672	39.1	1,583	36.6	1,985	41.3	6,519	38.2
Live together	2,010	54.6	2,173	50.8	2,224	51.4	2,328	48.5	8,735	51.1
Not living together	392	10.6	434	10.1	517	12.0	490	10.2	1,832	10.7
**Had sex last 4 weeks**										
Yes	1,922	52.2	2,230	52.1	2,709	62.7	2,877	59.9	9,737	57.0
No	1,760	47.8	2,049	47.9	1,615	37.3	1,925	40.1	7,349	43.0
**Visited by health worker**										
Yes	415	11.3	553	12.9	455	10.5	465	9.7	1,888	11.0
No	3,267	88.7	3,726	87.1	3,869	89.5	4,337	90.3	15,199	89.0
**FP message**										
Yes	3,190	86.6	3,743	87.5	3,807	88.1	4,254	88.6	14,993	87.7
No	492	13.4	536	12.5	517	11.9	549	11.4	2,093	12.3
**Desire to postpone**										
Yes	1,175	31.9	1,502	35.1	1,513	35.0	1,867	38.9	6,057	35.4
No	2,506	68.1	2,777	64.9	2,811	65.0	2,936	61.1	11,029	64.6
	Mean /SD	Median	Mean /SD	Median	Mean /SD	Median	Mean /SD	Median	Mean /SD	Median
**Parity**	2.5/2.2	2.1	2.4/2.3	2.0	2.4/2.2	2.0	2.2/2.3	1.7	2.4/2.3	1.9
**Distance**	2.1/2.7	1.4	1.8/1.6	1.3	1.6/2.1	1.2	1.7/2.3	1.2588	1.8/2.2	1.3
**Total**	3,682	100.0	4,279	100.0	4,323	100.0	4,802	100.0	17,086	100.0
Note: Sample of females 15 to 49 years of age. See Table 1 for variable definitions.										

Table 2d: Sample Characteristics for Uganda PMA Rounds 1-4

Indicator	Round									
	**1**		**2**		**3**		**4**		**Total**	
	No.	%	No.	%	No.	%	No.	%	No.	%
**Modern contraceptive use**										
Yes	754	21.0	939	26.3	939	25.9	1,025	27.5	3,657	25.2
No	2,838	79.0	2,628	73.7	2,691	74.1	2,705	72.5	10,861	74.8
**Residence**										
Rural	2,859	79.6	2,810	78.8	2,874	79.2	2,981	79.9	11,524	79.4
Urban	521	14.5	528	14.8	519	14.3	514	13.8	2,082	14.3
Metropolitan	212	5.9	228	6.4	237	6.5	236	6.3	912	6.3
**Education**										
No education	493	13.7	344	9.6	357	9.8	340	9.1	1,533	10.6
Primary school	724	20.2	742	20.8	752	20.7	790	21.2	3,008	20.7
Secondary school	2,375	66.1	2,480	69.6	2,522	69.5	2,600	69.7	9,977	68.7
**Wealth quintile**										
Poorest	654	18.2	607	17.0	675	18.6	716	19.2	2,651	18.3
Poorer	661	18.4	660	18.5	675	18.6	693	18.6	2,688	18.5
Middle	702	19.6	746	20.9	729	20.1	754	20.2	2,931	20.2
Richer	771	21.5	751	21.1	755	20.8	759	20.4	3,037	20.9
Richest	803	22.4	802	22.5	797	21.9	808	21.7	3,211	22.1
**Last child died**										
Yes	79	2.2	63	1.8	88	2.4	102	2.7	332	2.3
No	3,512	97.8	3,503	98.2	3,542	97.6	3,628	97.3	14,186	97.7
**Age group**										
15-19 years	754	21.0	802	22.5	742	20.4	767	20.5	3,064	21.1
20-24 years	757	21.1	759	21.3	834	23.0	790	21.2	3,140	21.6
25-29 years	678	18.9	610	17.1	686	18.9	639	17.1	2,612	18.0
30-34 years	490	13.6	470	13.2	467	12.9	532	14.3	1,959	13.5
35-39 years	392	10.9	396	11.1	373	10.3	415	11.1	1,576	10.9
40-44 years	314	8.7	313	8.8	317	8.7	333	8.9	1,276	8.8
45-49 years	207	5.8	217	6.1	213	5.9	255	6.8	892	6.1
**Cohabitation**										
Not married	1,268	35.3	1,288	36.1	1,255	34.6	1,213	32.5	5,025	34.6
Live together	1,993	55.5	1,941	54.4	2,082	57.4	2,115	56.7	8,131	56.0
Not live together	330	9.2	337	9.5	293	8.1	403	10.8	1,362	9.4
**Had sex last 4 weeks**										
Yes	1,914	53.3	1,985	55.7	2,164	59.6	2,254	60.4	8,317	57.3
No	1,677	46.7	1,581	44.3	1,466	40.4	1,476	39.6	6,200	42.7
**Visited by health worker**										
Yes	607	16.9	588	16.5	599	16.5	582	15.6	2,375	16.4
No	2,985	83.1	2,978	83.5	3,032	83.5	3,148	84.4	12,143	83.6
**FP message**										
Yes	2,929	81.5	2,849	79.9	2,893	79.7	3,021	81.0	11,691	80.5
No	663	18.5	717	20.1	738	20.3	709	19.0	2,827	19.5
**Desire to postpone**										
Yes	989	27.5	1,097	30.8	1,081	29.8	1,156	31.0	4,323	29.8
No	2,602	72.5	2,469	69.2	2,550	70.2	2,574	69.0	10,195	70.2
	Mean /SD	Median	Mean /SD	Median	Mean /SD	Median	Mean /SD	Median	Mean /SD	Median
**Parity**	3.0/2.9	2.3	2.8/2.8	2.0	2.8/2.8	2.1	3.0/2.8	2.4	2.9/2.8	2.2
**Distance**	2.7/3.3	1.5	2.6/2.9	1.5	3.7/7.8	1.4	2.7/3.5	1.40151	2.9/4.8	1.4
**Total**	3,591	100.0	3,566	100.0	3,630	100.0	3,730	100.0	14,518	100.0
Note: Sample of females 15 to 49 years of age. See Table 1 for variable definitions.										

Table 2e: Sample Characteristics for Burkina Faso PMA Rounds 3-4

Indicator	Round					
	3		4		Total	
	No.	%	No.	%	No.	%
**Modern contraceptive use**						
Yes	698	21.6	699	21.9	1,398	21.7
No	2,539	78.4	2,492	78.1	5,031	78.3
**Residence**						
Rural	2,401	74.2	2,386	74.8	4,787	74.5
Urban	430	13.3	414	13.0	844	13.1
Metropolitan	406	12.5	392	12.3	798	12.4
**Schooling**						
No education	2,036	62.9	2,058	64.5	4,094	63.7
Primary school	574	17.7	514	16.1	1,088	16.9
Secondary school	627	19.4	620	19.4	1,247	19.4
**Wealth tertile**						
Poorest	1,159	35.8	1,105	34.6	2,264	35.2
Middle	984	30.4	1,039	32.6	2,022	31.5
Richest	1,094	33.8	1,047	32.8	2,142	33.3
**Last child died**						
Yes	91	2.8	75	2.3	166	2.6
No	3,146	97.2	3,116	97.7	6,262	97.4
**Age group**						
15-19 years	774	23.9	694	21.8	1,468	22.8
20-24 years	585	18.1	549	17.2	1,134	17.6
25-29 years	582	18.0	556	17.4	1,138	17.7
30-34 years	422	13.0	464	14.5	886	13.8
35-39 years	393	12.1	405	12.7	799	12.4
40-44 years	263	8.1	293	9.2	557	8.7
45-49 years	218	6.7	229	7.2	447	6.9
**Cohabitation**						
Not married	821	25.4	781	24.5	1,602	24.9
Live together	2,125	65.7	2,137	67.0	4,263	66.3
Not live together	291	9.0	273	8.5	564	8.8
**Had sex last 4 weeks**						
Yes	1,835	56.7	1,824	57.1	3,659	56.9
No	1,402	43.3	1,367	42.9	2,769	43.1
**Visited by health worker**						
Yes	482	14.9	629	19.7	1,110	17.3
No	2,755	85.1	2,562	80.3	5,318	82.7
**FP message**						
Yes	1,991	61.5	1,959	61.4	3,950	61.4
No	1,246	38.5	1,232	38.6	2,478	38.6
**Desire to postpone**						
Yes	1,285	39.7	1,257	39.4	2,541	39.5
No	1,952	60.3	1,935	60.6	3,887	60.5
	Mean/SD	Median	Mean/SD	Median	Mean/SD	Median
**Parity**	2.5/2.6	1.8	2.9/2.7	2.3	2.7/2.7	2.0
**Distance**	3.8/8.0	1.3	2.8/3.5	1.4	3.3/6.2	1.4
**Total**	3,237	100.0	3,191	100.0	6,428	100.0
Note: Sample of females 15 to 49 years of age. See Table 1 for variable definitions.						

Table 3: Direct estimates of the modern contraceptive prevalence rate and 95% uncertainty intervals in Ghana, Ethiopia, Kenya, Uganda and Burkina Faso by round

Country	Region	Round 1			Round 2			Round 3			Round 4			Change: round 1 to 4
		Mean	Lower	Upper	Mean	Lower	Upper	Mean	Lower	Upper	Mean	Lower	Upper	
Ghana	Ashanti	16.1	13.7	18.7	18.3	15.7	21.2	17.1	14.6	19.8	23.7	21.1	26.4	7.6
	Brong-Ahafo	17.4	13.9	21.3	16.2	12.4	20.7	22.8	18.5	27.6	24.3	19.9	29.2	7.0
	Central	16.0	12.4	20.1	23.1	18.3	28.4	21.8	18.3	25.5	25.8	22.6	29.1	9.8
	Eastern	13.5	10.4	17.2	12.3	9.0	16.4	18.8	14.4	23.9	24.4	19.8	29.4	10.9
	Greater-Accra	15.0	12.6	17.7	15.9	13.1	19.0	19.9	17.2	22.9	22.8	20.1	25.8	7.8
	Northern	7.7	5.4	10.6	5.6	3.5	8.4	10.3	7.3	13.8	13.3	10.2	16.9	5.5
	Upper-East	19.3	13.7	25.9	17.1	11.6	23.7	16.1	11.9	21.2	32.4	27.5	37.5	13.1
	Upper-West	26.5	18.7	35.6	19.6	13.0	27.8	24.7	19.0	31.2	34.7	28.2	41.6	8.1
	Volta	8.8	5.8	12.6	7.1	4.4	10.8	15.5	10.8	21.2	15.7	11.4	20.9	6.9
	Western	6.0	3.7	9.1	7.2	4.7	10.6	13.9	10.7	17.6	12.1	9.0	15.8	6.0
	**ALL**	**14.0**	**12.9**	**15.1**	**14.4**	**13.2**	**15.5**	**18.1**	**17.0**	**19.3**	**22.8**	**21.7**	**24.0**	**8.8**
Ethiopia	Addis Ababa	20.6	16.7	25.0	22.8	18.3	27.8	29.1	24.3	34.1	27.0	22.3	32.1	6.4
	Afar	3.0	0.1	13.9	6.5	1.4	17.4	24.9	14.3	38.4	13.2	6.3	23.3	10.2
	Amhara	35.9	33.1	38.8	35.6	33.0	38.3	31.8	29.5	34.2	35.3	32.9	37.8	-0.6
	Benishangul Gumuz	10.9	4.3	21.7	11.4	5.5	20.1	16.2	9.3	25.4	14.9	8.4	23.9	4.0
	Dire Dawa	12.5	0.8	45.3	28.5	8.0	58.8	29.3	12.2	52.2	37.3	19.0	58.8	24.8
	Ethiopia Somali	7.5	0.9	24.3	5.9	0.7	19.7	6.1	1.0	18.3	6.7	1.3	19.1	-0.8
	Gambella	23.2	10.0	41.8	23.4	12.1	38.4	24.4	12.2	40.6	24.7	12.1	41.6	1.5
	Harari	28.3	11.4	51.4	20.2	5.3	45.6	21.2	7.5	42.4	22.4	7.4	45.5	-5.9
	Oromiya	18.6	16.9	20.3	21.1	19.5	22.8	23.0	21.0	25.1	21.8	19.8	23.8	3.2
	SNNPR	23.7	21.2	26.3	22.2	20.1	24.5	27.6	25.4	29.8	28.0	25.9	30.2	4.3
	Tigray	20.1	16.2	24.4	22.9	18.8	27.3	22.9	19.1	27.0	22.1	18.4	26.2	2.0
	**ALL**	**23.4**	**22.3**	**24.6**	**24.4**	**23.3**	**25.5**	**26.8**	**25.6**	**27.9**	**27.0**	**25.9**	**28.2**	**3.6**
Kenya	Bungoma	43.9	38.2	49.7	37.1	32.1	42.2	45.3	40.3	50.4	43.5	38.7	48.5	-0.3
	Kericho	39.8	35.4	44.5	37.8	33.7	42.1	42.9	38.7	47.1	38.9	35.2	42.8	-0.9
	Kiambu	43.4	38.6	48.2	48.4	43.7	53.1	44.9	40.5	49.4	50.0	45.6	54.4	6.6
	Kilifi	27.9	23.6	32.4	25.9	22.1	30.0	32.0	27.9	36.3	31.4	27.7	35.2	3.5
	Kitui	39.8	34.9	44.8	39.6	35.2	44.1	51.0	46.6	55.3	53.1	48.9	57.3	13.3

	Nairobi	45.3	41.6	49.0	41.4	37.9	45.0	51.3	47.6	54.9	48.6	45.1	52.2	3.3
	Nandi	44.6	39.2	50.2	44.5	39.7	49.3	50.1	45.2	55.1	47.6	42.9	52.4	3.0
	Nyamira	50.0	44.1	56.0	52.2	46.6	57.9	58.5	52.9	64.0	56.7	51.2	62.0	6.6
	Siaya	41.9	36.7	47.3	40.1	35.4	44.9	45.0	40.1	50.0	50.0	45.4	54.6	8.1
	**ALL**	**41.7**	**40.1**	**43.3**	**40.3**	**38.8**	**41.8**	**46.5**	**45.0**	**48.0**	**46.0**	**44.5**	**47.4**	**4.3**
Uganda	Central1	25.8	21.6	30.3	35.1	30.5	39.9	34.8	30.1	39.7	29.2	25.0	33.7	3.5
	Central2	18.9	14.9	23.6	25.9	21.1	31.1	32.3	27.3	37.6	30.6	26.0	35.5	11.7
	East_Central	17.3	13.8	21.3	21.6	17.8	25.7	27.1	23.1	31.5	26.5	22.4	30.9	9.2
	Eastern	19.4	16.3	22.8	26.4	22.9	30.2	21.9	18.6	25.5	27.3	23.9	31.0	7.9
	Kampala	29.1	23.1	35.7	38.2	31.9	44.8	38.9	32.6	45.4	34.0	28.0	40.4	4.9
	Karamoja	10.7	5.8	17.7	4.5	1.6	9.8	4.7	1.6	10.2	3.9	1.1	9.6	-6.8
	North	24.3	19.8	29.2	27.1	22.6	31.9	23.0	18.9	27.5	25.8	21.4	30.5	1.5
	South_West	24.5	20.5	28.9	25.2	21.2	29.5	24.0	19.9	28.5	32.9	28.2	37.9	8.4
	West_Nile	7.1	4.5	10.5	14.5	10.5	19.3	12.3	8.6	16.9	17.3	13.1	22.2	10.3
	Western	26.5	22.5	30.9	30.9	26.4	35.6	27.8	23.8	32.2	29.8	25.6	34.2	3.2
	**ALL**	**21.0**	**19.7**	**22.4**	**26.3**	**24.9**	**27.8**	**25.9**	**24.5**	**27.3**	**27.5**	**26.1**	**29.0**	**6.5**
Burkina Faso	Boucle du Mouhoun							18.6	14.9	22.9	20.0	16.2	24.1	1.3
	Cascades							26.5	19.9	34.0	19.1	13.3	26.2	-7.3
	Centre							30.3	25.9	35.0	36.1	31.3	41.1	5.8
	Centre Est							11.8	7.8	17.1	18.5	13.1	24.9	6.6
	Centre Nord							18.8	14.7	23.6	15.1	11.3	19.7	-3.7
	Centre Ouest							23.2	18.5	28.4	20.6	16.1	25.7	-2.6
	Centre Sud							27.7	19.5	37.2	29.8	20.2	40.9	2.0
	Est							17.7	14.2	21.7	23.6	19.1	28.6	5.9
	Haut Bassins							28.0	23.0	33.3	30.9	25.8	36.4	2.9
	Nord							21.1	15.5	27.8	18.8	14.2	24.1	-2.3
	Plateau Central							26.0	18.1	35.3	14.6	9.0	22.0	-11.4
	Sahel							12.7	8.7	17.8	10.3	6.7	14.9	-2.4
	Sud Ouest							19.1	12.4	27.3	14.7	8.6	22.7	-4.4
	**ALL**							**21.6**	**20.2**	**23.0**	**21.9**	**20.5**	**23.4**	**0.3**

Note: Lower and upper denote the boundaries of the 95% uncertainty interval for direct estimate

Covariate selection

Effective prediction depends on striking a balance between bias and stability. A saturated model can reduce bias but at the cost or risk of unstable predictions; a prediction based on too few covariates, or their transforms, is relatively stable but at the cost or risk of increased bias. This is particularly true in our study given sampling and measurement errors in the covariates. Our selection criteria for covariates are based on: (1) theory; (2) a review of previous empirical studies; and (3) model assessment. We also use a deviance information criterion (DIC) in selecting several variables and specifying their definitions. For example, the literature indicates that survival status of previous births influences women's contraceptive use.[1] There are two possible ways to measure previous child survival--the number of children who have died or whether the last child born in the preceding two years is still alive. The model with the latter measurement showed a smaller DIC and therefore was used in the study. Based on these criteria, we arrived at a list of 12 covariates: residence, schooling, wealth quintile, child survival, age, cohabitation, recent sex, health worker visit, family planning message, fertility intention, parity, and distance to the nearest facility. See the appendix for their definitions (p 3)

Accounting for survey weight

While there are a variety of approaches for accommodating survey sampling weights in a frequentist analysis, including reciprocal propensity weighting (e.g. the Horvitz-Thompson approach) and case-specific propensity as a covariate, the latter is most directly implementable in a Markov chain Monte Carlo context.[2,3] The goal is to build a model wherein the sampling process is "ignorable", i.e., that the analysis includes all variables that affect the probability of a person being included in the sample, and thereby accommodate the weights when estimating the fixed effects.

We evaluated the impact of including sampling weight as a model covariate. Including both a linear and quadratic terms did not appreciably change the population estimates and predictions compared to non-inclusion. Therefore, in the spirit of parsimony, our estimates are based on models that do not include the

sampling weight as a covariate. We account for sampling weight in the post-estimation aggregation from individual level to EA-level, regional, and national estimates.

Model checking and assessment

We use several methodological approaches to assess the predictive performance of our model. Within-sample assessments are more optimistic than out-of-sample ones, because the former use the same data for fitting and evaluation. Technical adjustments are not generally available in this complex modeling situation. For example, if we wish to perform a chi-square test for the overall standardized residual of the model, it is difficult to determine the degrees of freedom.

The most important indicator in model diagnosis is the model residual, which is defined as the difference between the MLE and Bayesian estimates. The model residual is then standardized to eliminate the influence of level and scale and provides a metric, i.e. the standardized residual, which is comparable across models. The values and distribution of the standardized residual indicate whether the model captures the most important covariates and whether the model assumptions (e.g. structure of random effects) are reasonable. The standardized residual is based on the direct estimate and thus measures its difference from a model-based estimate. This approach is satisfactory if the direct estimates have a nearly Gaussian distribution.

However, the mCPR outcome in this study does not have a Gaussian distribution, and therefore we have to develop a new diagnostic measure, Z-value. It indicates the percentile location of the direct estimate in amongst its predictive distribution from the BHM. In general, the Z-value is less dependent on the Gaussian assumption, and it will be identical to the standardized residual if the predictive distribution is exactly Gaussian. In this study the Z-value is more appropriate than the standardized residual because the

mCPR values for many small areas are very low and could be far from following a Gaussian distribution. See webappendix (pp) for additional details.

Z-value vs Avelogistic plots

Figures 1a-1e illustrates the distribution of Z-values versus avelogistic estimates for the four countries where each data point is an area-round (e.g. region or county 1 in round 1; region or county 1 in round 2) because the model uses area-level random effects. The data points in the right panel represent specific EA-rounds because our study interest is regional estimates. The lack of a pattern in the left panel's graphs indicates that our model performs equally well across low, middle and high-mCPR areas.

Priors for model parameters

The following priors are used in the study. And the information has been added to the appendix.

$$\beta_i \sim N(0{,}10000), i = 1, \dots, NX$$
$$u[t,j] \sim \begin{cases} N(0, precu[1,j]), & t = 1; \quad j = 1, \dots, NEA \\ N(mu[t,j], precu[t,j]), & t = 2, \dots, NT; j = 1, \dots, NEA \end{cases}$$
$$mu[t,j] \sim \rho * u[t-1,j], t = 2, \dots, NT; j = 1, \dots, NEA$$
$$precu[t,j] \sim \begin{cases} 1/\tau^2, & t = 1; \quad j = 1, \dots, NEA \\ 1/((1-\rho^2)\tau^2), & t = 2, \dots, NT; j = 1, \dots, NEA \end{cases}$$
$$\rho \sim Uniform(0.01{,}0.99)$$
$$\tau^2 \sim Gamma(0.001{,}0.001)$$

where NX denotes the number of coefficients; NT denotes the number of rounds; NEA denotes the number of EAs.

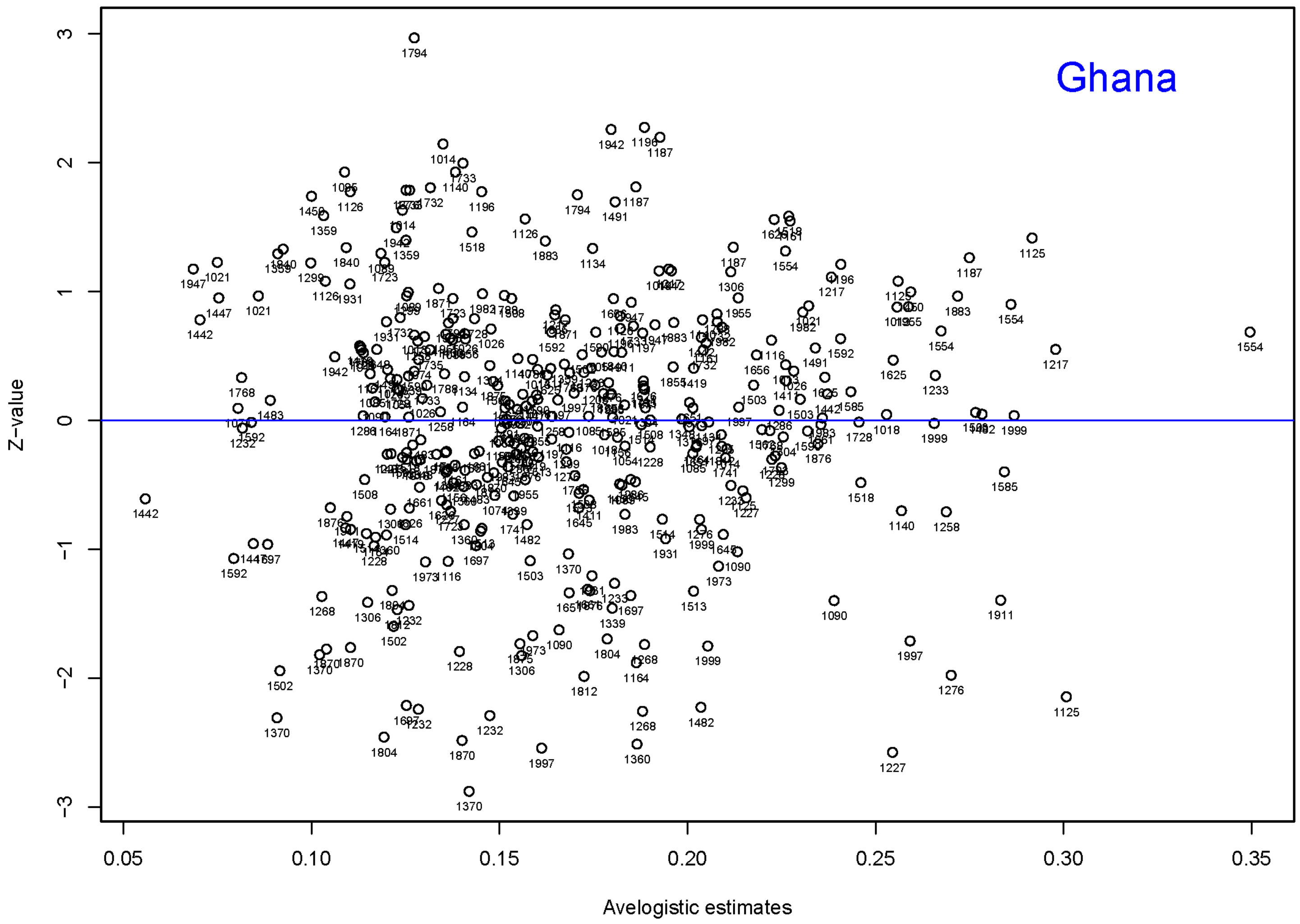

Figure 1a: Z-value vs. Avelogistic estimates: Ghana round 1-4

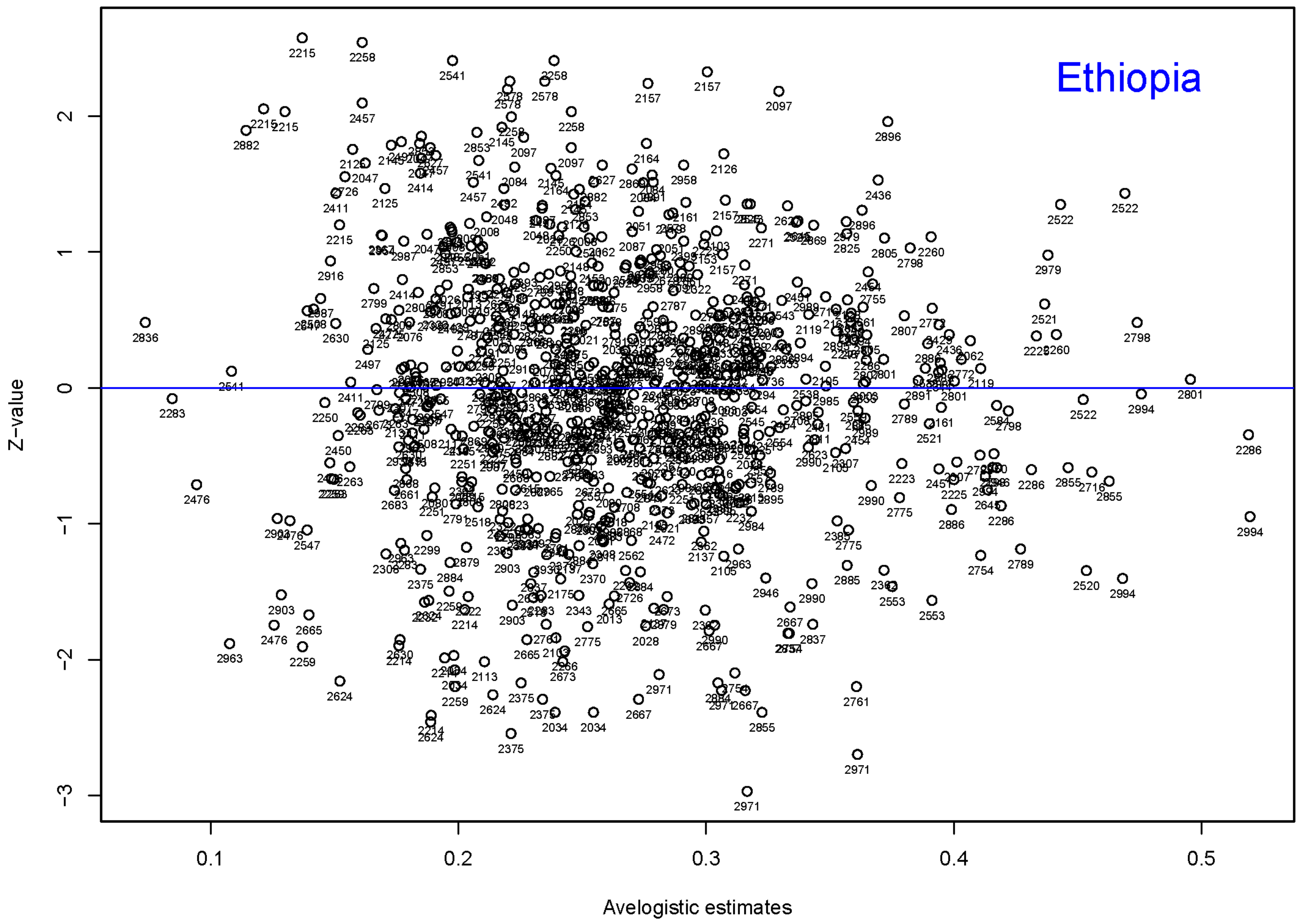

Figure 1b: Z-value vs. Avelogistic estimates: Ethiopia round 1-4

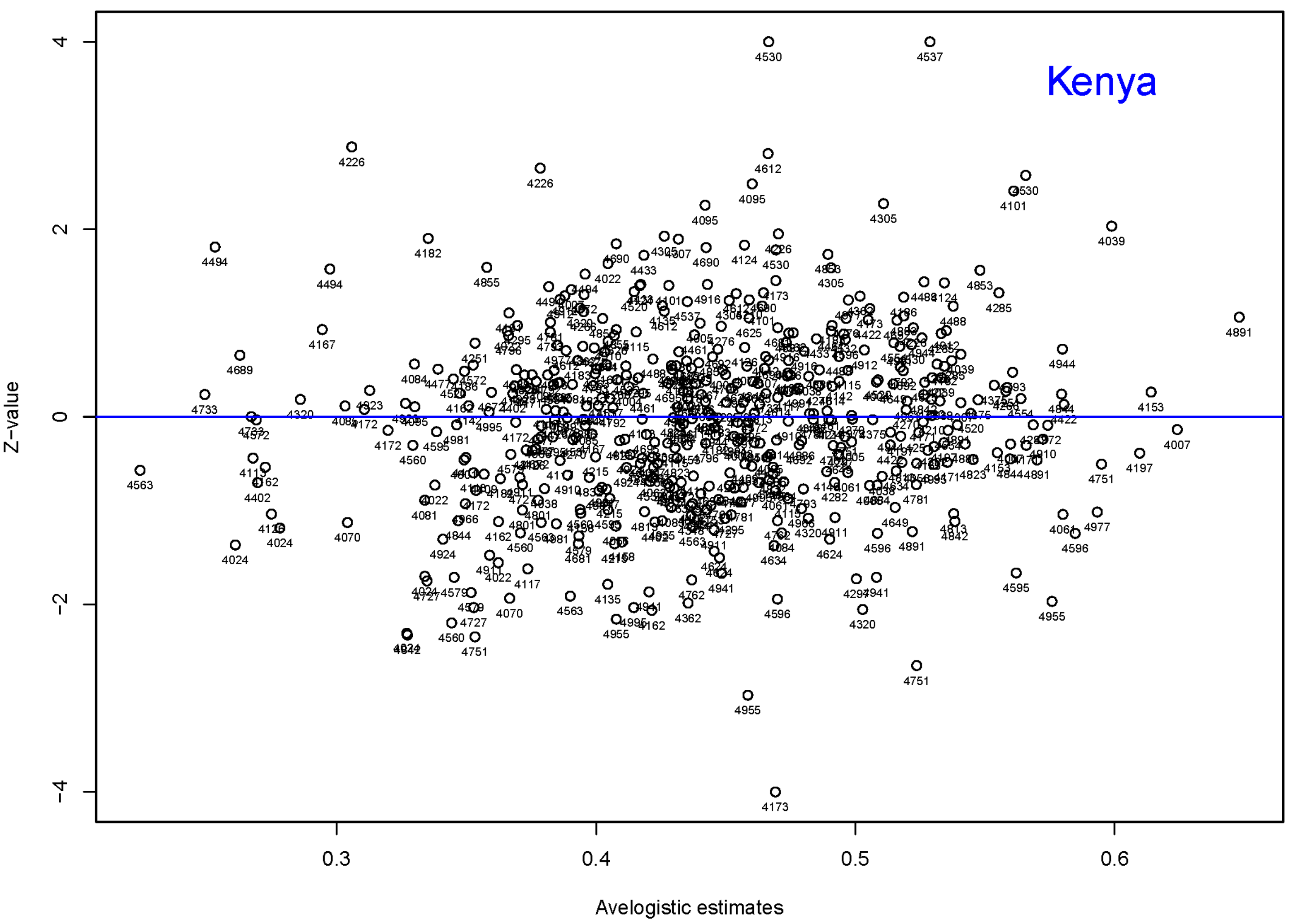

Figure 1c: Z-value vs. Avelogistic estimates: Kenya round 1-4

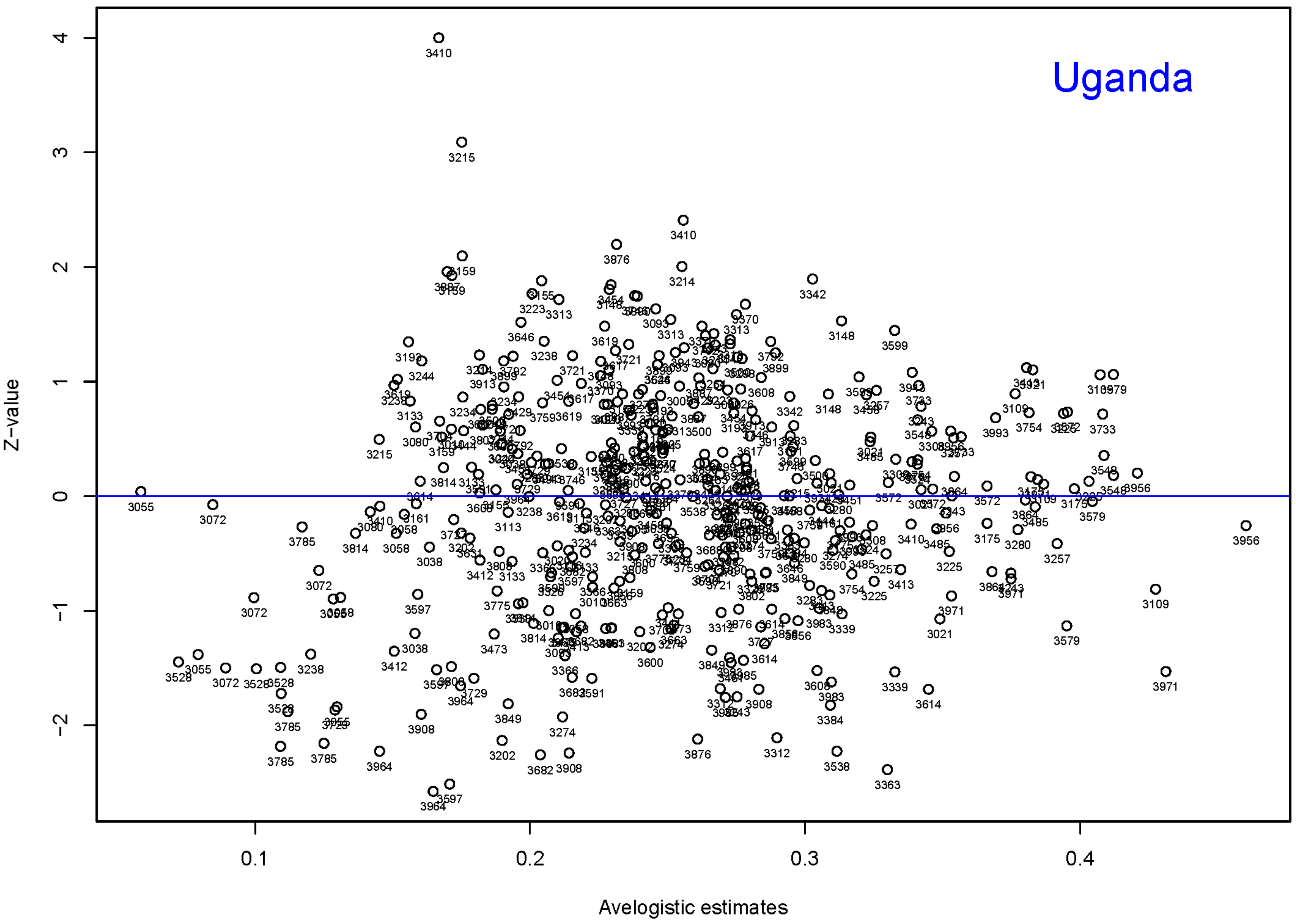

Figure 1d: Z-value vs. Avelogistic estimates: Uganda round 1-4

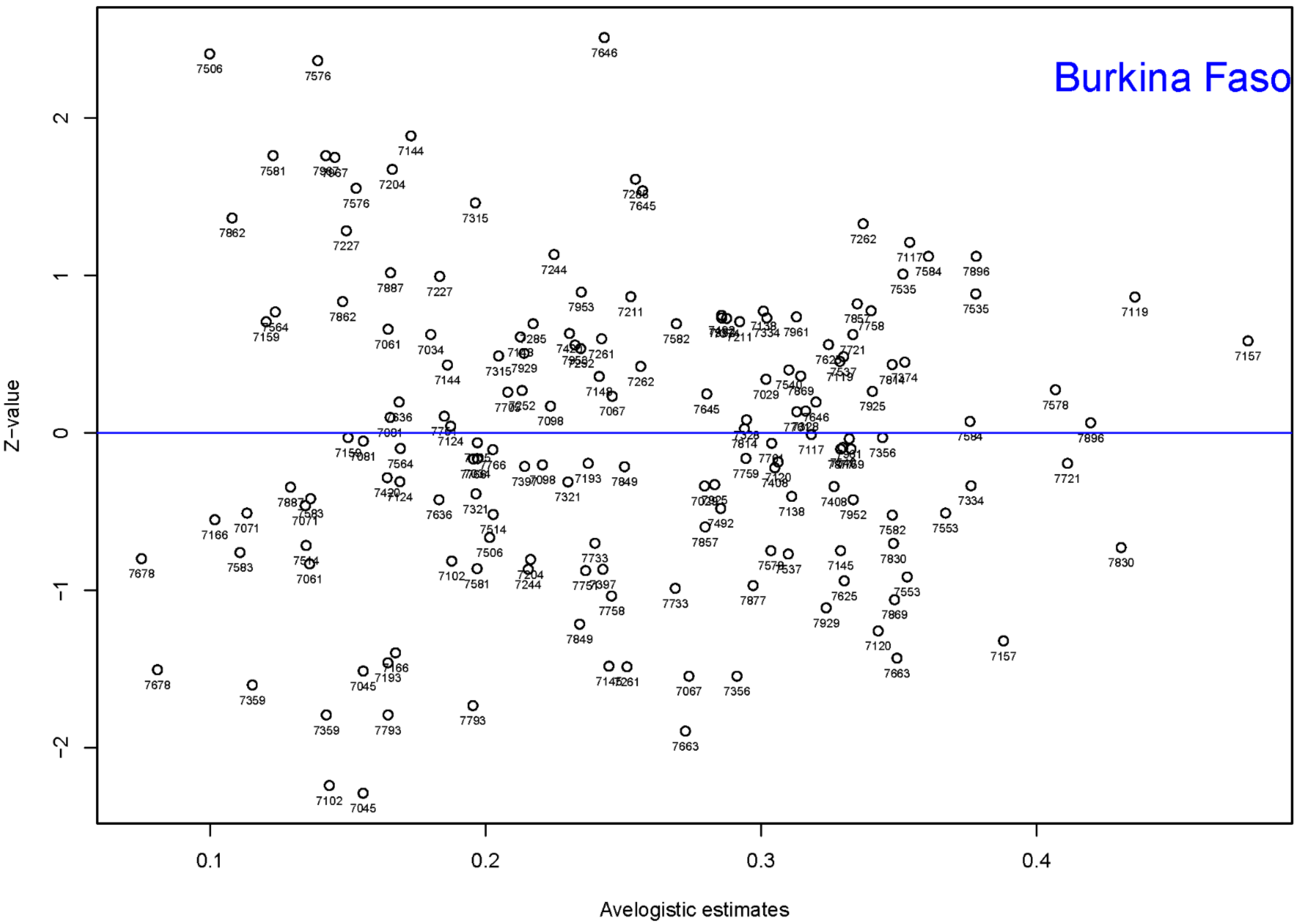

Figure 1e: Z-value vs. Avelogistic estimates: Burkina Faso round 3-4

Shrinkage plots

In a shrinkage plot, each line denotes a subnational unit (region or county) in a survey round with the standard deviations of the direct estimates as whiskers; the middle line shows the direct estimates; and the bottom shows the predicted estimates from the BHM. The model shrinks the direct estimates toward the model-based predictions, with the shrinkage mainly determined by the uncertainty of the direct estimates.

Figures 2a-2e show the shrinkage after removing the influence of a logistic regression. Instead of using a separate logistic model, our indicator, called avelogistic, is based on a logistic model with the same set of covariates averaged over the posterior distribution of coefficients and random effects of the BHM. The shrinkage of the residual between direct-minus-logistic estimates toward the residual between Bayesian-minus-avelogistic estimates indicate the extent to which Bayesian estimates help achieve a balance between information from women's direct report of contraceptive use (i.e. direct estimates) and pure model-based prediction (i.e. avelogistic estimates). The balance is largely determined by the accuracy of the direct estimates, where estimates with a longer whiskers tend to shrink more, and have larger variation in random effects. The plots show that our BHMs are achieving a balance between direct and pure model-based estimates.

Table 2: Median and 95% uncertainty intervals (UI) of parameter estimates from Bayesian hierarchical models

Parameters	Burkina Faso			Ethiopia			Ghana			Kenya			Uganda		
	Median	95% UI		Median	95% UI		Median	95% UI		Median	95% UI		Median	95% UI	
Intercept	-4.25	-4.66	-3.89	-5.09	-5.40	-4.81	-4.49	-4.86	-4.12	-4.45	-4.75	-4.13	-4.42	-4.72	-4.08
Round 2				0.07	-0.05	0.19	0.04	-0.15	0.24	0.00	-0.12	0.12	0.29	0.15	0.44
Round 3				0.16	0.03	0.29	0.41	0.20	0.65	0.22	0.08	0.36	0.25	0.09	0.41
Round 4	-0.04	-0.24	0.17	0.16	0.02	0.30	0.64	0.42	0.88	0.29	0.15	0.43	0.32	0.13	0.48
Residence (ref=rural)	0.82	0.48	1.17	0.79	0.47	1.13	-0.02	-0.36	0.30	0.17	-0.05	0.38	0.25	-0.06	0.59
Metropolitan	0.96	0.59	1.39	0.83	0.32	1.28	0.38	-0.07	0.84	0.25	-0.11	0.60	0.61	0.29	0.95
Primary (ref=no education)	0.53	0.34	0.71	0.26	0.16	0.37	0.39	0.24	0.53	0.99	0.77	1.20	0.56	0.39	0.75
Secondary	0.79	0.59	1.00	0.17	0.04	0.30	0.54	0.35	0.72	1.13	0.90	1.34	0.70	0.52	0.92
Last child died	-0.88	-1.39	-0.37	-0.70	-1.00	-0.41	-0.18	-0.68	0.28	-0.68	-1.03	-0.34	-0.42	-0.73	-0.11
Parity	0.25	0.20	0.29	0.17	0.14	0.19	0.22	0.19	0.25	0.19	0.17	0.22	0.12	0.10	0.15
Poorer (ref=poorest)				0.11	-0.06	0.28	0.19	0.02	0.36	0.12	0.00	0.22	0.30	0.14	0.47
Middle	0.00	-0.23	0.23	0.21	0.02	0.39	0.13	-0.07	0.33	0.13	0.01	0.25	0.38	0.20	0.57
Richer				0.37	0.17	0.58	0.17	-0.06	0.39	0.21	0.06	0.36	0.43	0.26	0.62
Richest	0.12	-0.16	0.39	0.35	0.13	0.59	-0.04	-0.30	0.21	0.11	-0.07	0.30	0.50	0.30	0.72
20-24 years (ref=15-19)	0.89	0.63	1.15	0.93	0.79	1.07	0.97	0.79	1.15	1.13	0.98	1.28	0.76	0.61	0.92
25-29 years	0.69	0.42	0.96	0.88	0.73	1.03	0.89	0.69	1.09	1.51	1.36	1.68	0.92	0.76	1.11
30-34 years	0.54	0.23	0.85	0.56	0.39	0.73	0.63	0.42	0.84	1.37	1.19	1.55	0.88	0.68	1.08
35-39 years	-0.02	-0.37	0.34	0.13	-0.05	0.31	0.40	0.16	0.64	1.20	1.00	1.37	0.82	0.61	1.05
40-44 years	-0.16	-0.56	0.24	-0.22	-0.44	-0.01	0.03	-0.23	0.30	0.69	0.48	0.89	0.61	0.38	0.87
45-49 years	-1.07	-1.55	-0.57	-1.20	-1.47	-0.93	-0.69	-1.00	-0.38	-0.14	-0.36	0.09	-0.03	-0.31	0.25
Live together (ref=no married)	-0.02	-0.23	0.21	0.79	0.67	0.91	-0.14	-0.28	0.00	0.57	0.47	0.68	-0.01	-0.13	0.12
Not live together	0.23	-0.06	0.52	0.77	0.60	0.95	0.04	-0.11	0.19	0.72	0.58	0.85	0.12	-0.04	0.29
Had sex last 4 weeks	1.49	1.33	1.65	1.91	1.80	2.02	1.22	1.11	1.32	1.22	1.13	1.31	1.07	0.96	1.19
Visited by health worker	0.43	0.25	0.61	0.40	0.31	0.49	0.59	0.46	0.72	0.30	0.18	0.42	0.29	0.17	0.41
FP message	0.34	0.16	0.50	0.35	0.25	0.44	0.24	0.12	0.36	0.14	0.01	0.26	0.20	0.07	0.33

Desire to postpone	-0.02	-0.16	0.13	0.21	0.12	0.29	0.12	0.01	0.23	0.27	0.17	0.36	0.19	0.08	0.30
Distance	-0.02	-0.05	0.00	0.00	-0.01	0.01	0.00	-0.01	0.01	0.00	-0.02	0.03	-0.01	-0.02	0.01
rho	0.42	0.09	0.69	0.92	0.88	0.95	0.75	0.65	0.84	0.83	0.74	0.90	0.77	0.65	0.87
tau2	0.39	0.26	0.55	0.87	0.69	1.08	0.68	0.52	0.87	0.33	0.26	0.43	0.38	0.28	0.51

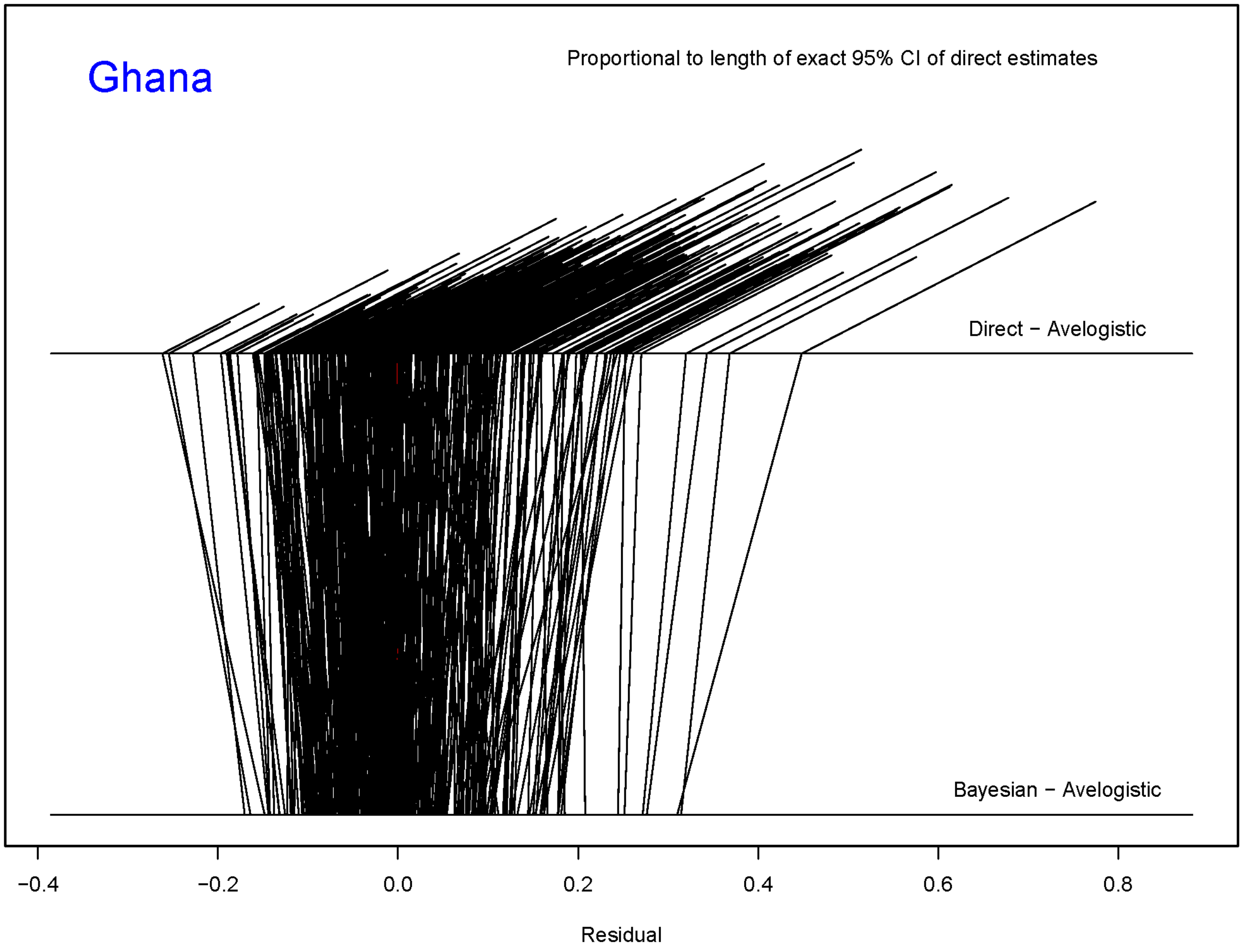

Figure 2a: Residual shrinkage: Ghana round 1-4

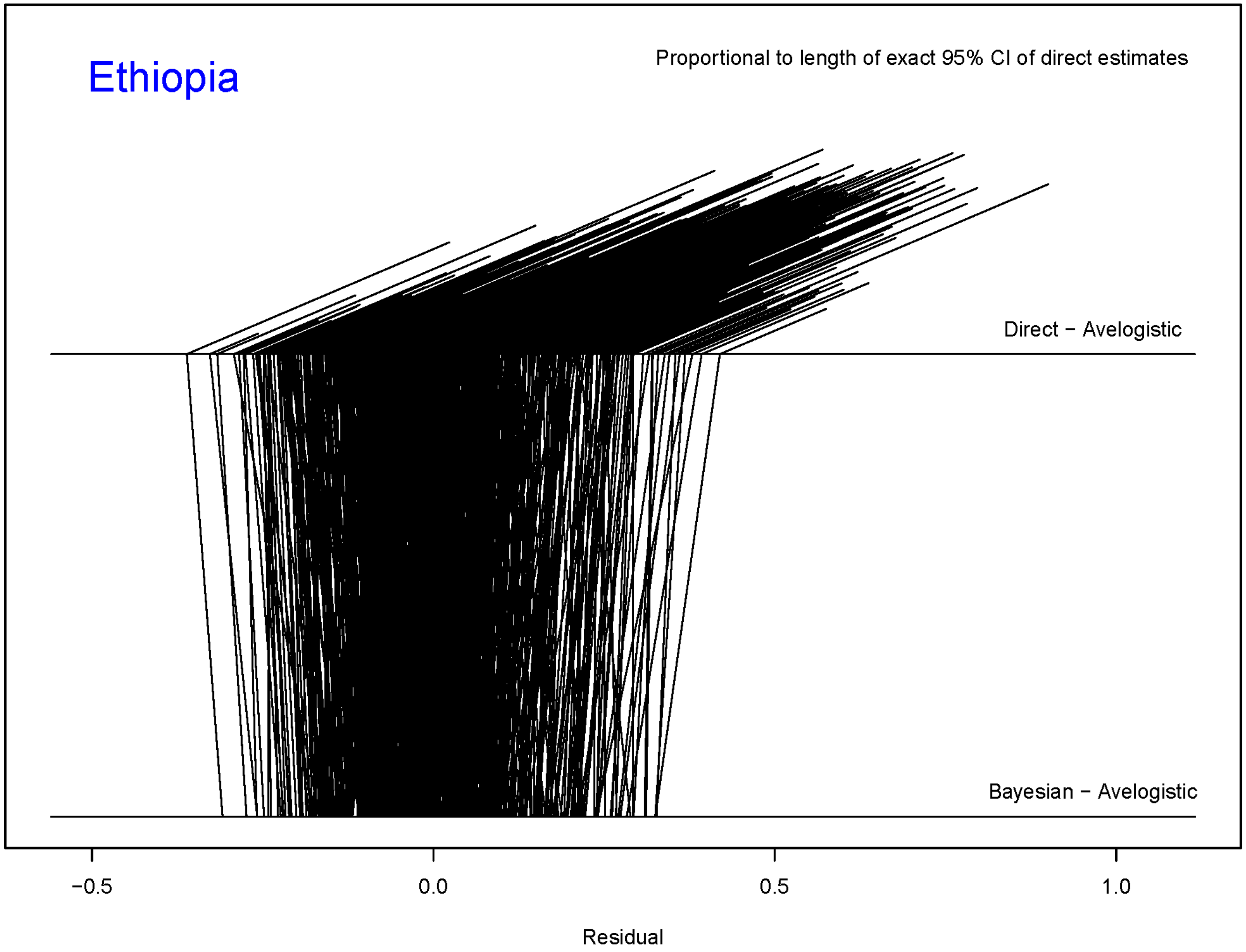

Figure 2b: Residual shrinkage: Ethiopia round 1-4

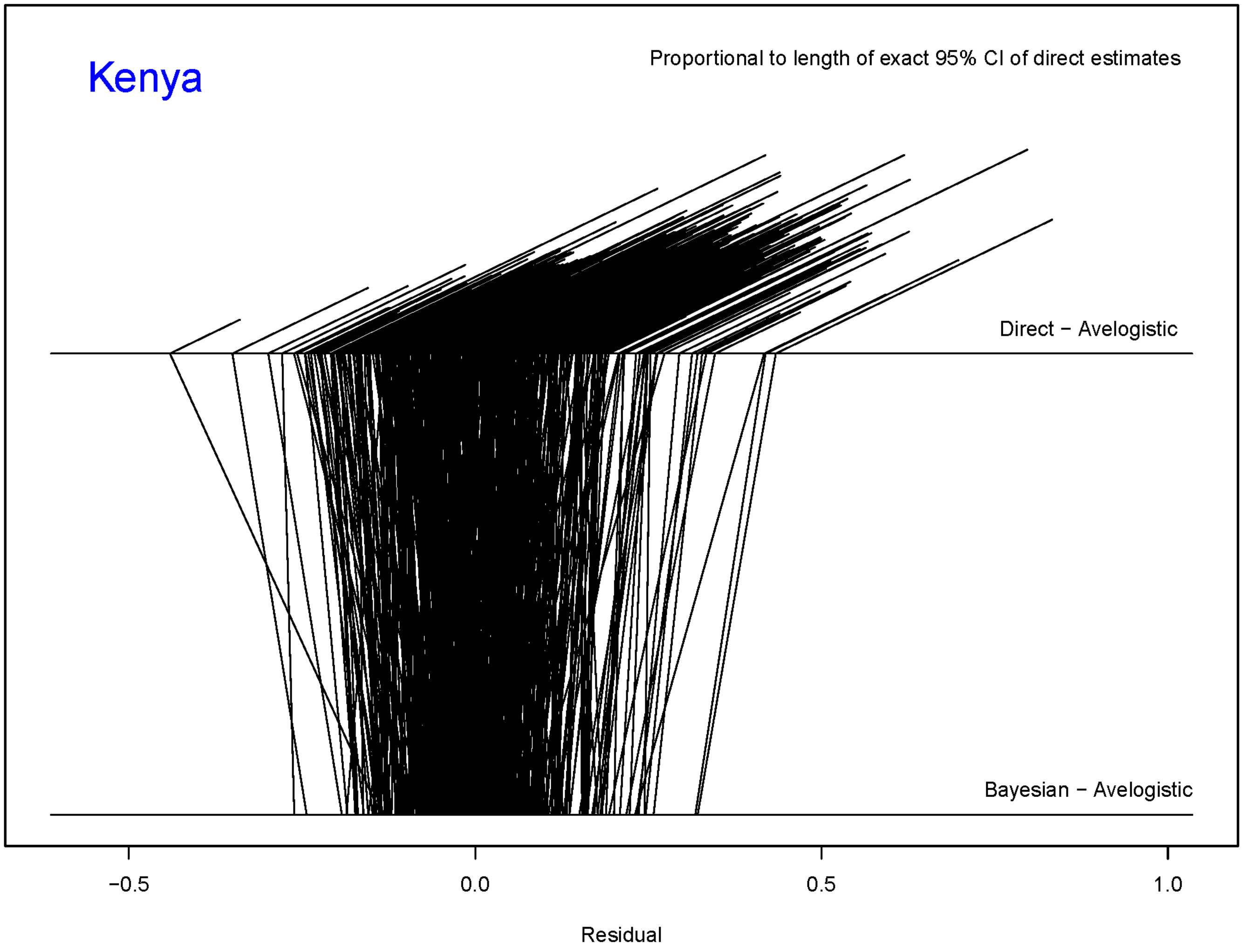

Figure 2c: Residual shrinkage: Kenya round 1-4

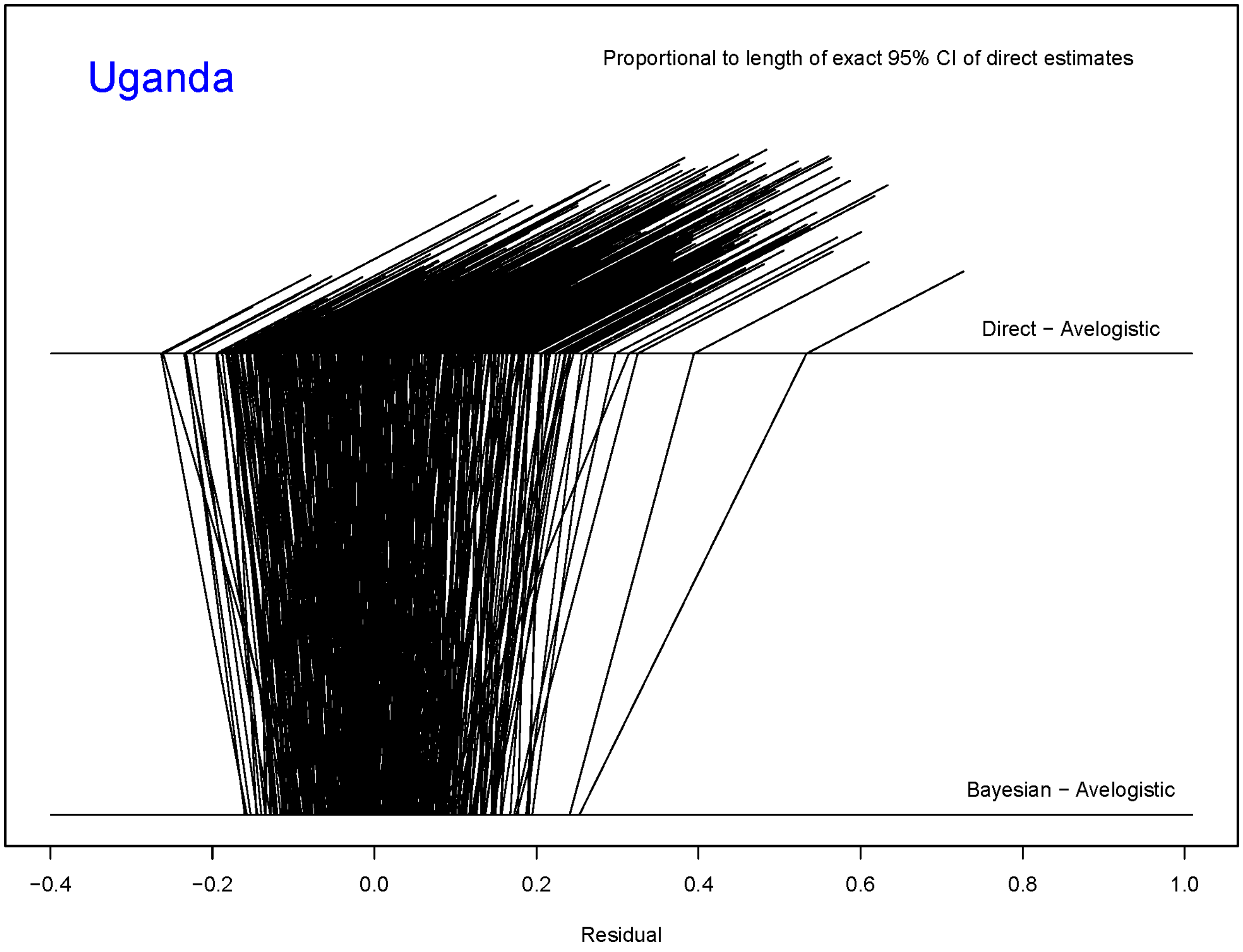

Figure 2d: Residual shrinkage: Uganda round 1-4

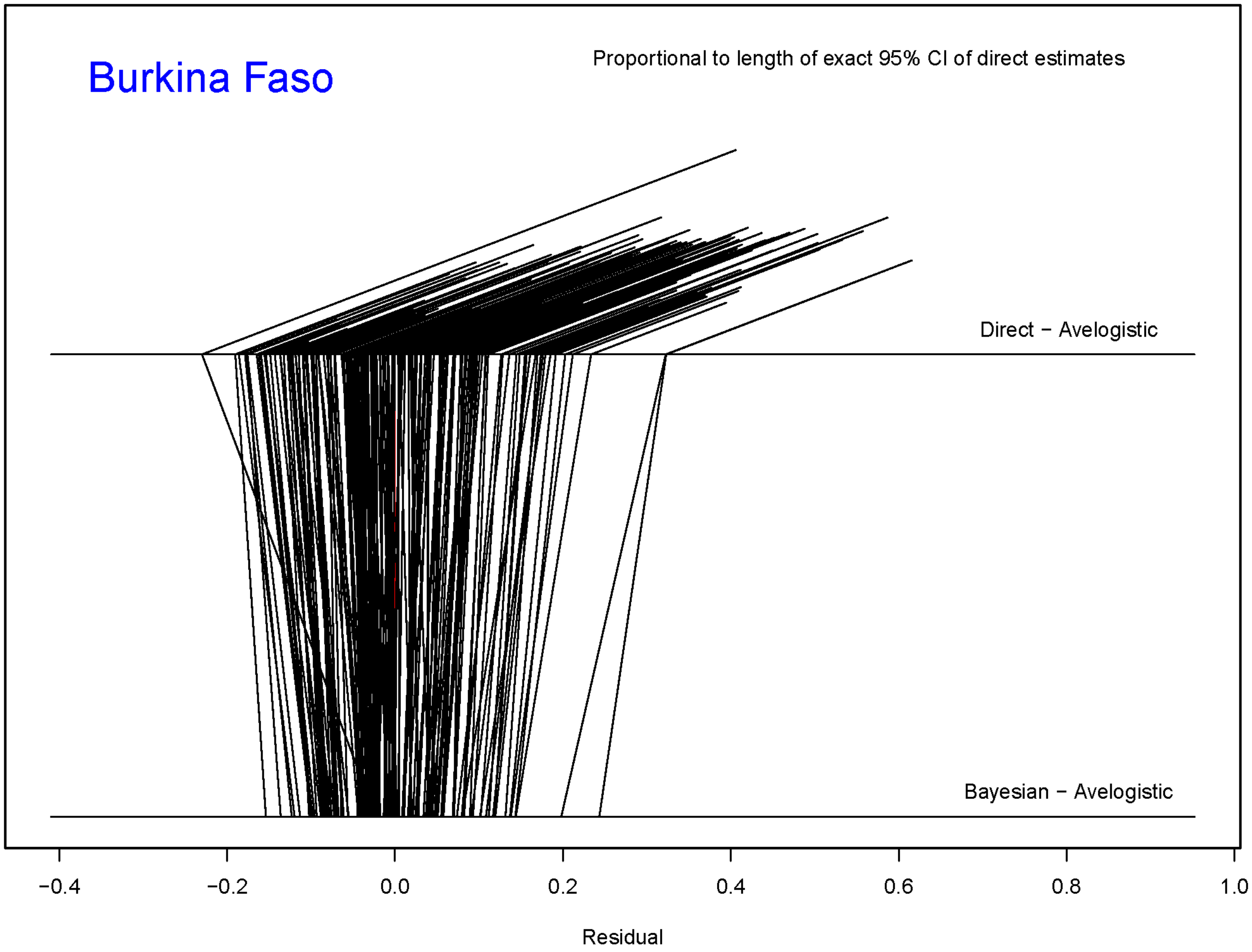

Figure 2e: Residual shrinkage: Burkina Faso round 3-4

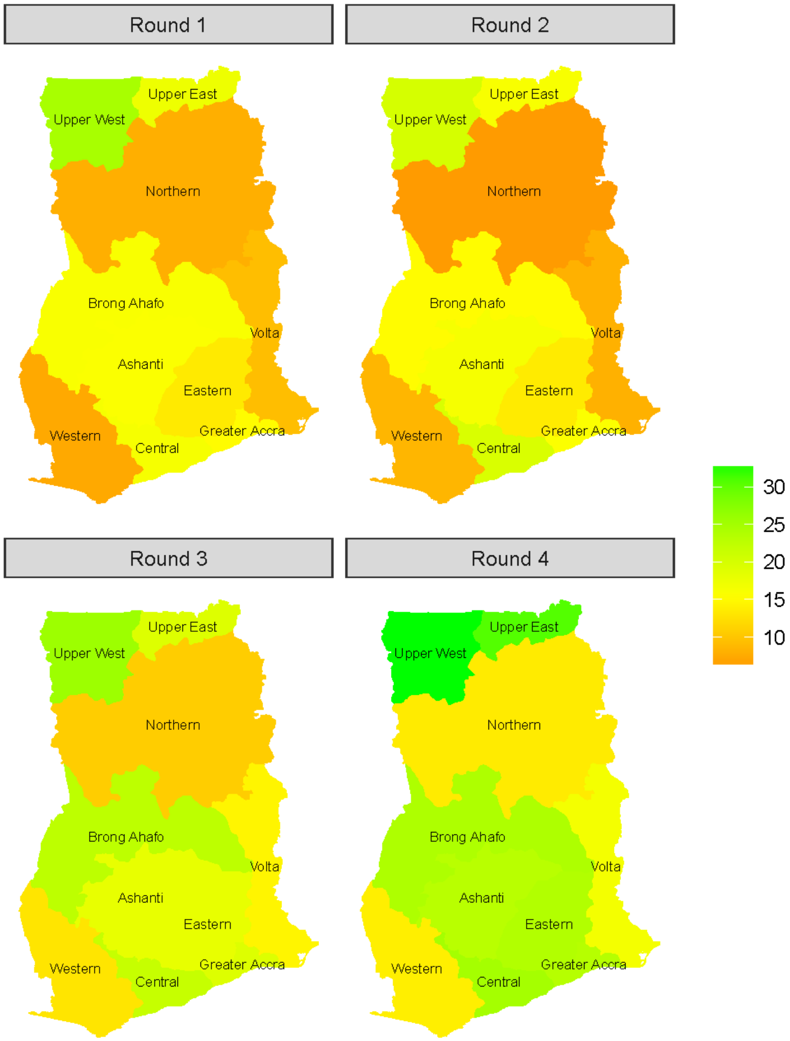

Figure 3a: Temporal and geographic variation in Bayesian estimates over four rounds of PMA2020 survey in Ghana

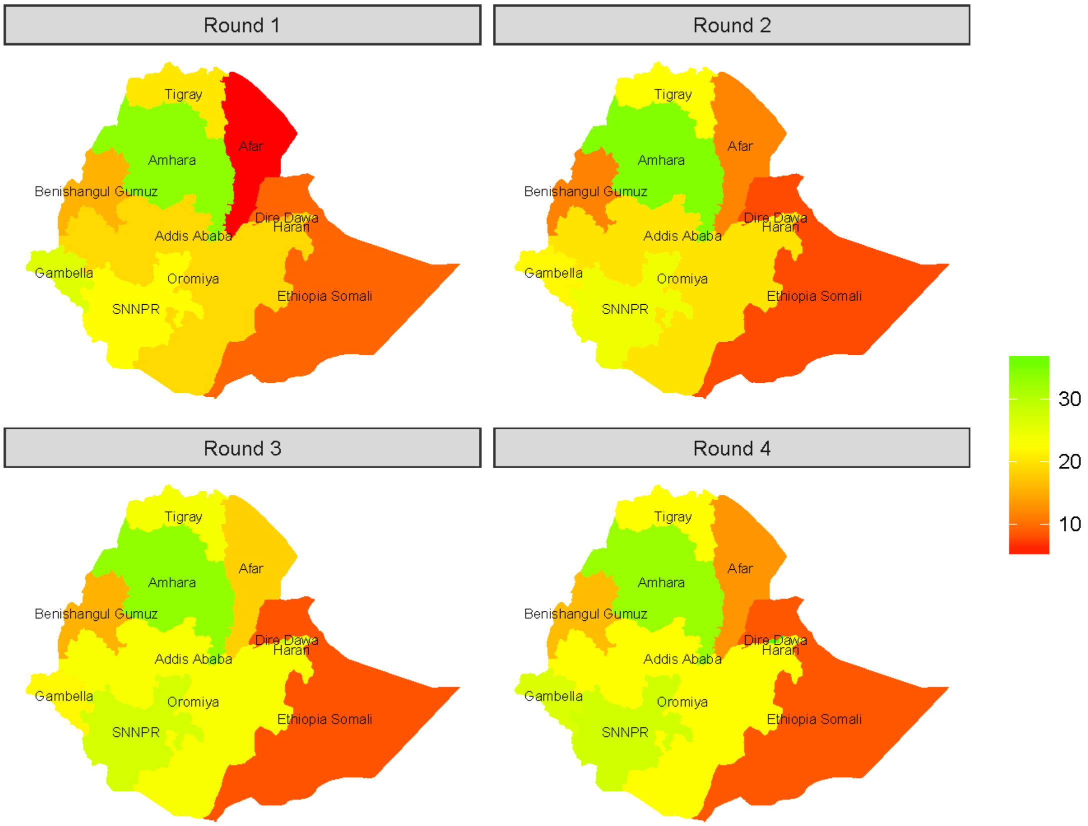

Figure 3b: Temporal and geographic variation in Bayesian estimates over four rounds of PMA2020 survey in Ethiopia

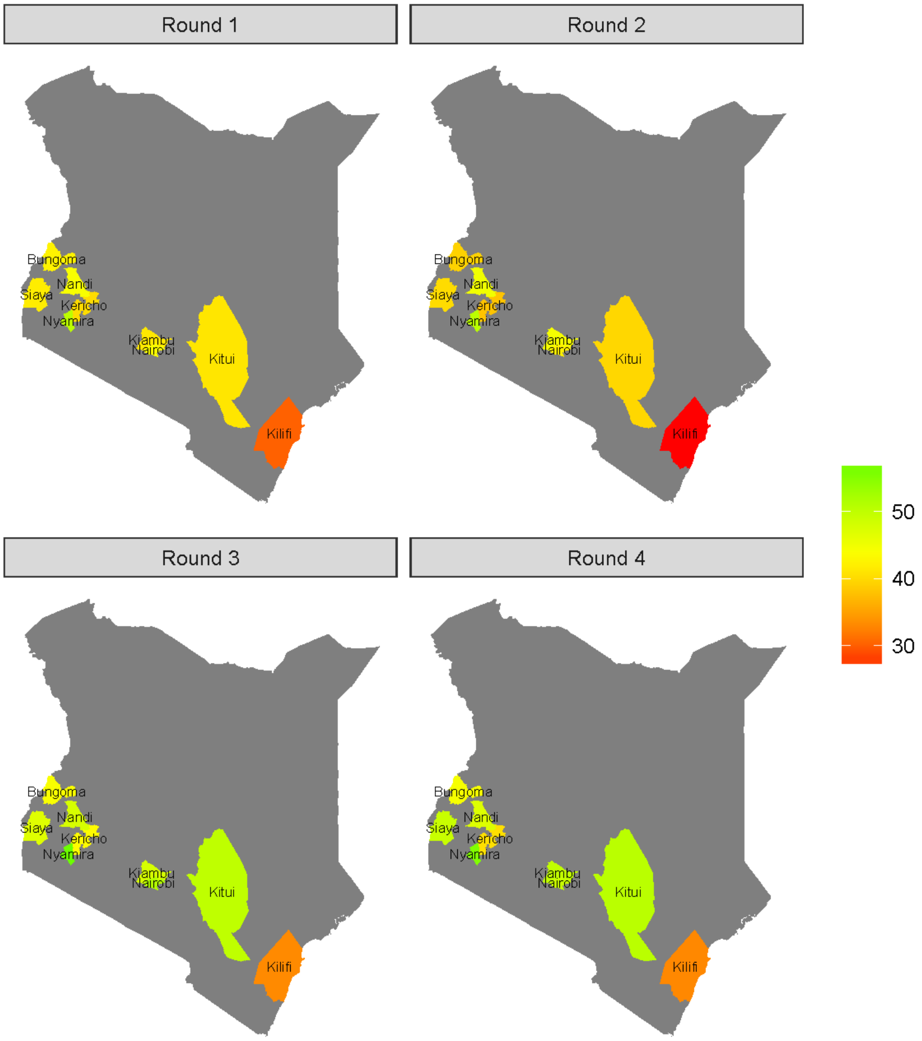

Figure 3c: Temporal and geographic variation in Bayesian estimates over four rounds of PMA2020 survey in Kenya

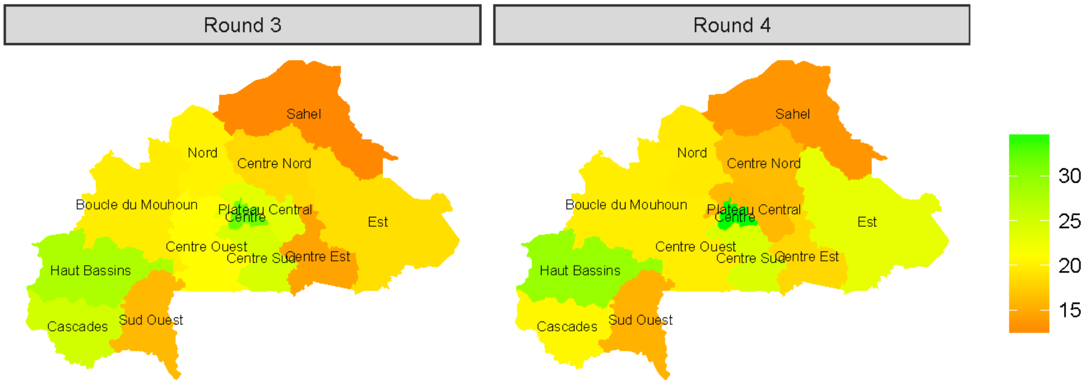

Figure 3d: Temporal and geographic variation in Bayesian estimates over two rounds of PMA2020 survey in Burkina Faso

References

1. Lloyd CB, Ivanov S. The effects of improved child survival on family planning practice and fertility. *Stud Fam Plann* 1988; **19**(3): 141-61.
2. Chen C, Wakefield J, Lumely T. The use of sampling weights in Bayesian hierarchical models for small area estimation. *Spatial and spatio-temporal epidemiology* 2014; **11**: 33-43.
3. Vandendijck Y, Faes C, Kirby RS, Lawson A, Hens N. Model-based inference for small area estimation with sampling weights. *Spatial Statistics* 2016; **18, Part B**: 455-73.
